# The structural chemistry and biosynthesis of chlorophylls

**DOI:** 10.1039/d6cb00082g

**Published:** 2026-06-25

**Authors:** C. Neil Hunter, Felix S. Morey-Burrows

**Affiliations:** a School of Biosciences, University of Sheffield Firth Court, Western Bank Sheffield S10 2TN UK c.n.hunter@sheffield.ac.uk

## Abstract

Chlorophylls (Chls) harvest the solar energy that drives photosynthesis, which underpins most of the food chains on our planet. Starting from protoporphyrin IX, just seven biosynthetic reactions culminate in the synthesis of Chl *a*, the major light-absorbing pigment on Earth. Other such pigments, Chls *b*, *c*, *d* and *f*, widen the absorption range in the visible and red regions of the spectrum, and several bacteriochlorophylls (BChls), BChls *a*, *b* and *g* in particular, open new spectral windows allowing organisms to harvest near infra-red light. This perspective surveys the structural features of porphyrins, chlorins and bacteriochlorins that impart their characteristic absorption features, then presents a similar analysis of the biosynthetic intermediates leading to Chls *a*, *b*, *c*, *d* and *f*. The interlinked Chl and BChl biosynthetic pathways are summarised, then the rest of the perspective focusses on the enzymes that synthesise Chls *a*, *b*, *c*, *d* and *f*. AlphaFold 3 was used to model a complete set of structures for Chl biosynthesis enzymes, predicting intersubunit associations and the arrangements of cofactors and bound substrates, and providing insights into catalytic mechanisms. A new scheme for binding substrates and transferring products between pathway enzymes suggests how synthetic biology approaches can assemble hybrid Chl and BChl pathways to expand the spectral range for harvesting and using solar energy.

## Abstract 2

A set of calculated structures for chlorophyll biosynthesis enzymes, and their possible clustering at the membrane surface to promote transfers of substrates and products.

## Graphical abstract

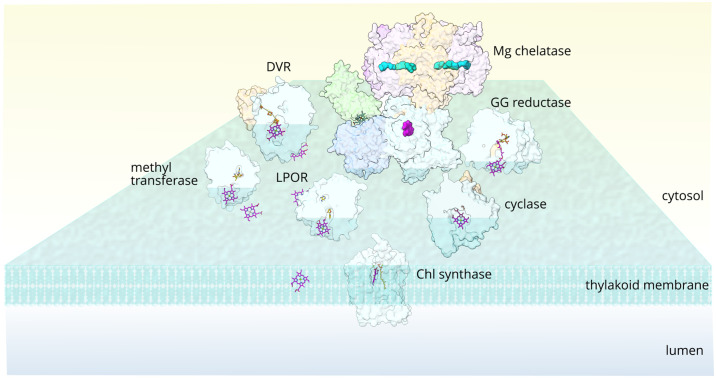

## 1. Introduction

Chlorophylls (Chls) harvest the sunlight that provides the main source of energy for the biosphere. Photosynthetic metabolism uses absorbed solar energy to drive a cascade of excitation energy, electron and proton transfers, transiently storing this energy as a proton motive force, and subsequently in a chemical form, adenosine triphosphate (ATP), which fuels cell growth and division. Photoautotrophic metabolism fixes carbon dioxide, forming reduced carbon compounds such as carbohydrates on a massive scale, and global primary productivity, estimated as 119 ± 12 Pg carbon yr^−1^,^1^ represents a vital, long-term store of solar energy for all life on Earth. Heterotrophs extract this energy from reduced carbon compounds by employing a series of catabolic reactions that also culminate in transfers of electrons and protons, formation of a proton-motive force, and the synthesis of ATP. Chl makes all this possible as “…the pigment that negotiates the cosmic gap between the light of the sun and life on Earth.”^2^

The Earth's Chl metabolism includes the annual terrestrial cycles of vernal Chl biosynthesis then autumnal breakdown, as well as the production of Chl *a* in the oceans. Although largely unseen, marine bacteria and algae synthesise Chl on a vast scale, with their rapid turnover measured on timescales of hours and days, and with each cell synthesising millions of Chl pigments. The huge accumulation of biomass on Earth,^3,4^ is attributable to the global production of Chls, mainly Chl *a*. It has been remarked that Chl biosynthesis is the only product of a biochemical pathway visible from outer space,^5^ and one can estimate that around 10^33^ Chl molecules are present on the land and in the seas, equivalent to an annual production of ∼10^9^ tonnes. Thus, global primary productivity rests on the seven enzymes that convert protoporphyrin IX (PPIX) to Chl *a*.

The purpose of this review is to conduct a comparative survey of the Chls and BChls, to summarise the catalytic steps leading to Chls *a*, *b*, *c*, *d* and *f*, and to present the structures of the enzymes of Chl biosynthesis. The functions of Chls, as essential pigments in photosynthetic light-harvesting and reaction center (RC) complexes, are outside the scope of this review, as is the pathway leading to the synthesis of PPIX. Some valuable articles on Chl biosynthesis were consulted for this review, for example.^6–9^ Also very useful are reviews on natural and synthetic Chls,^10^ a review of symmetry and chirality in Chls,^11^ and one wide-ranging review that includes rhodopsin-based energy transduction in marine bacteria.^12^ The regulation of Chl biosynthesis, reviewed in ref. 13, is crucial for the physiological role of these pigments and their assembly into photosynthetic complexes, but is not considered here. The motivation for this article is to bring together information on the structural chemistry and absorption properties of Chls and BChls. Furthermore, it aims to update the Chl biosynthesis field by including recent progress towards the understanding of mechanistic and structural aspects of Chl biosynthesis enzymes, some of which had eluded biochemical and structural analyses for decades. Specifically, we provide a complete structural overview of the Chl biosynthesis pathway, using the AlphaFold family of computational tools to augment existing structures of Chl biosynthesis enzymes. Finally, we consider what it means to have a biochemical pathway, how substrates and products might move between enzymes, and the future directions that research on Chl and BChl biosynthesis might take, by thinking of these pathways as biosynthetic modules. As such, they can be re-routed and combined to provide engineered photosynthetic organisms with a wider palette of pigments than those furnished by evolution. Such modules could be transplanted into heterotrophs with no previous history of making Chl,^14,15^ providing a foundation for designing and building new photosynthetic organisms.

## 2. The structures of porphyrins, chlorins and bacteriochlorins in relation to their biological function

**Fig. 1:**
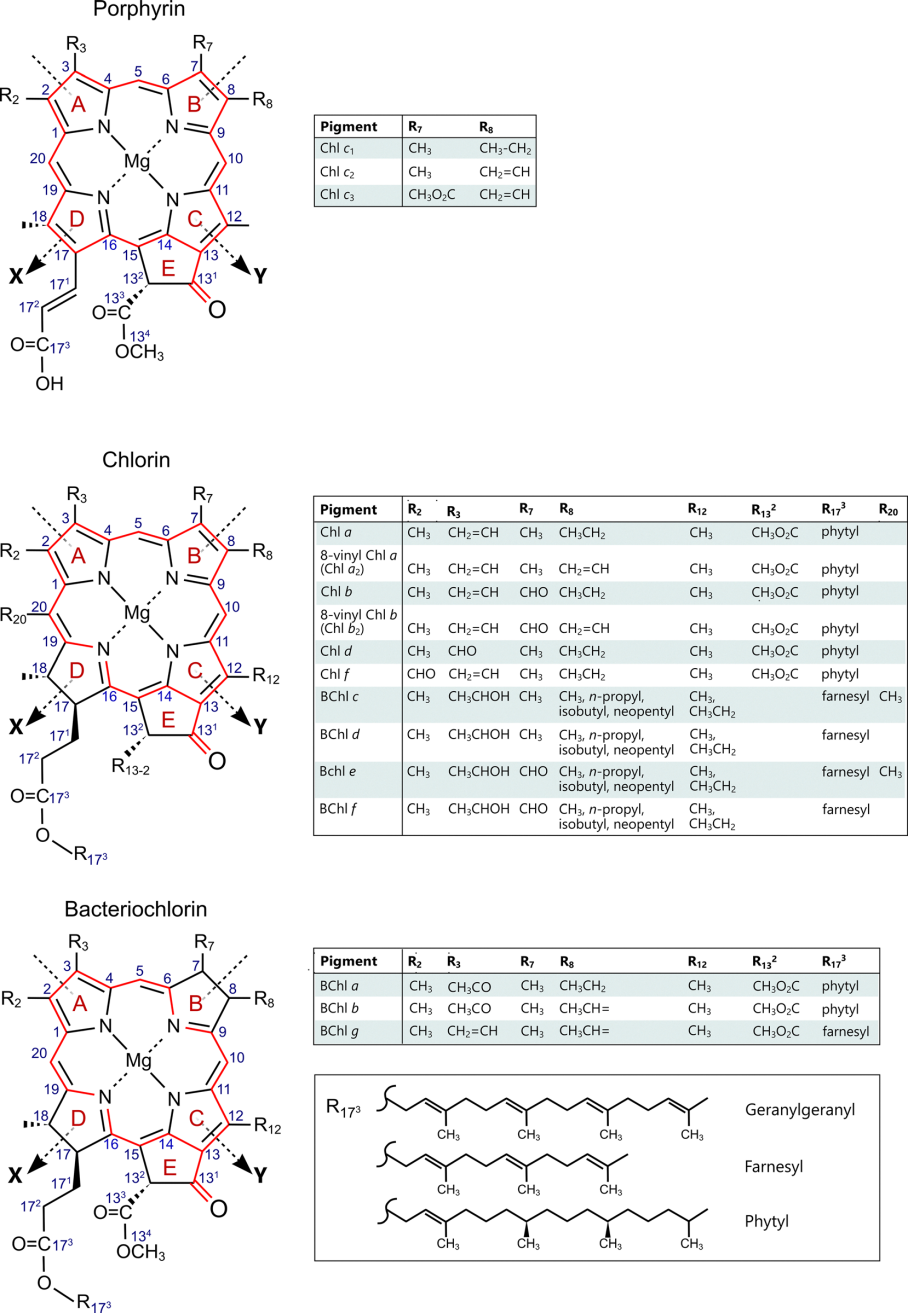
Structures of porphyrin-, chlorin-, and bacteriochlorin-type Chls and BChls. The red outlines indicate the extent of conjugation for each macrocycle. The arrows and *X*, *Y* labels denote the molecular axes that run between central pyrrole nitrogen atoms. Carbon atoms are numbered (in blue) according to standard IUPAC-IUB nomenclature. Exocyclic R groups for each pigment appear in the respective tables. Esterification at C17^3^ generally involves attachment of a phytyl group, with farnesyl used for some of the BChls. Geranylgeranyl (GG) is also included in the bottom table, because this is the esterifying group in the BChl *a* found in the purple phototroph *Rhodospirillum rubrum*.

This group of pigments consists of cyclic arrangements of aromatic bonds that form an extended π–electron system, all derived from a porphyrin macrocycle consisting of four pyrrole units connected to one another by methine bridges. Depending on the extent of oxidation of the macrocycle, these pigments can be classified as porphyrins, which are fully unsaturated; chlorins (17,18-dihydroporphyrins) in which ring D is reduced; and bacteriochlorins (7,8-17,18-tetrahydroporphyrins) with two reduced rings, B and D. These three types of tetrapyrrole are depicted in Fig. 1, which shows (in red outlines) how the reduction of one, then two rings progressively modifies the extent of conjugation of these π systems. One anomalous feature of the chlorin group is the presence of BChls *c*, *d*, *e* and *f*, which, despite their name, are actually chlorins, hence their listing in Fig. 1 along with Chls. The accompanying tables in Fig. 1 catalogue the wide variety of exocyclic groups that can further modify the optical properties of Chls and BChls.

**Fig. 2:**
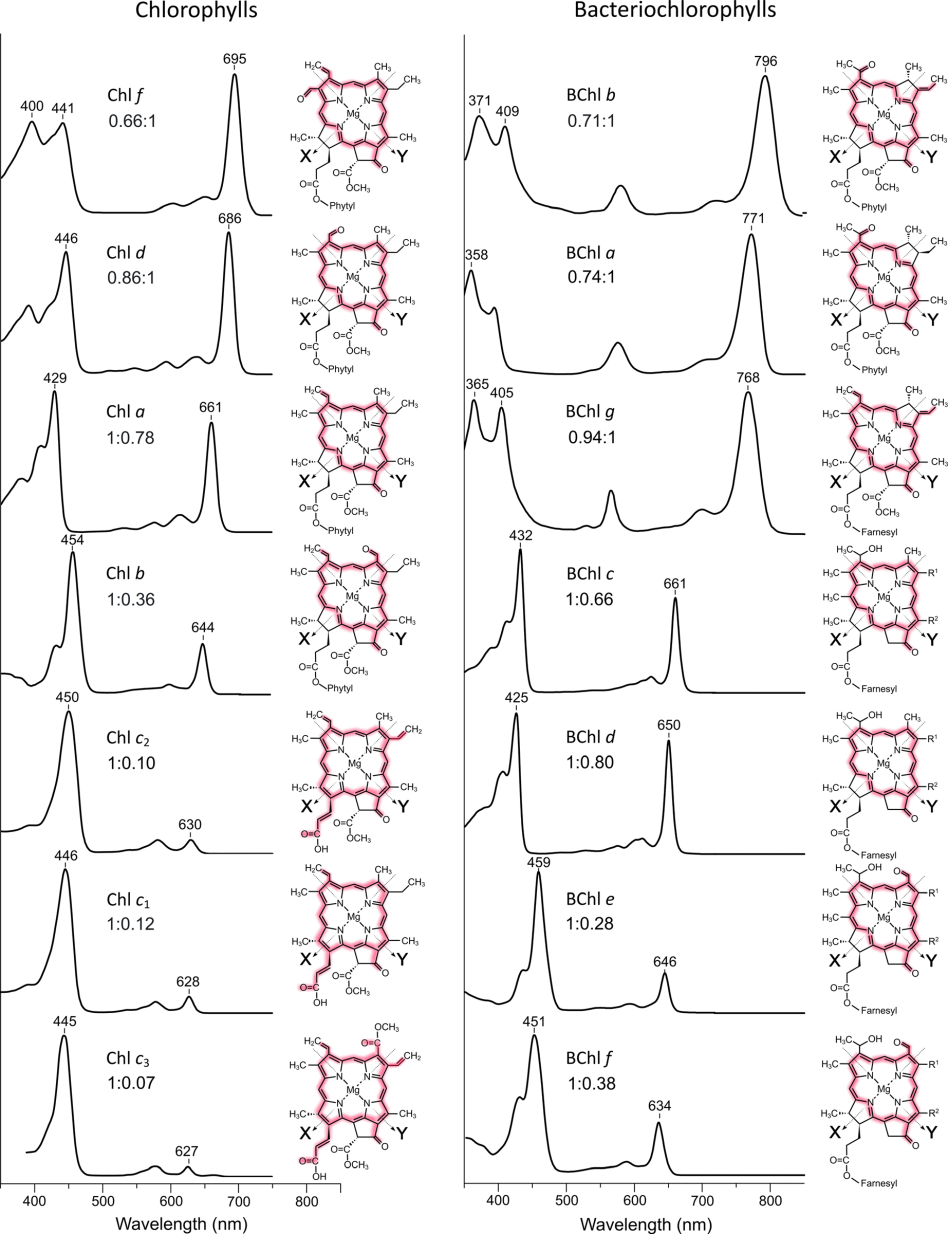
The absorption spectra of some Chls and BChls, with their respective chemical structures. The spectra of pigments in diethylether were downloaded from PhotochemCAD (https://www.photochemcad.com/databases/natural-chlorophylls;^16^). Ref. 246 also contains valuable data on the optical and structural properties of Chls and BChls. Chl and BChl spectra were replotted to facilitate stacking according to the position of the Q_*Y*_ absorption maximum, with the lowest energy Q_*Y*_ bands at the top of each stack. Diffuse red outlines illustrate macrocyle conjugation and its extension to exocyclic groups. The ratio of B-band : Q_*Y*_-band amplitudes at their respective absorption maxima are shown for each pigment. In nearly all cases this ratio correlates with the extent of the red shift of the Q_*Y*_ band. The 8V derivatives of Chls *a* and *b* are not included.

π-conjugated molecules of this size would be expected to absorb visible light and indeed porphyrins, Chls and BChls are coloured, with a strong B-band (Soret) absorption around 380–450 nm. Fig. 2 shows the absorption spectra of the porphyrin-type Chls *c*_1_, c_2_ and *c*_3_; the chlorins, Chls *a*, *b*, *d* and *f* and BChls *c*, *d*, *e* and *f*; and the bacteriochlorins, BChls *a*, *b* and *g*, all in solvent. The choice of solvent affects the absorption wavelength maxima, as shown by the spectra in PhotochemCAD,^16^ and diethyl ether was arbitrarily selected for the spectra in Fig. 2. Each spectrum is accompanied by a pigment structure, with diffuse red outlines used to provide a purely graphic representation of the extent of conjugation. The absorption spectra, which are arranged in two equal columns according to their Chl/BChl nomenclature, fall unevenly into two main spectroscopic groups – eleven with their lowest energy bands in the 630–700 nm region, and the three true BChls *a*, *b* and *g* further redshifted above 760 nm. Within the former group, only Chls *c*_1_, *c*_2_ and *c*_3_ retain some porphyrin symmetry. The spectra are stacked to show the progressive intensification and redshifting of the lowest energy absorption band, for the Chls (left) and BChls (right). The following sections briefly explain why the state of reduction of the macrocyle, and modification of conjugation by exocyclic conjugated groups, are functionally important for various classes of photosynthetic organisms, allowing them access to different spectral regions of the solar energy that reaches the Earth.

### 2.1. Porphyrins

**Fig. 3:**
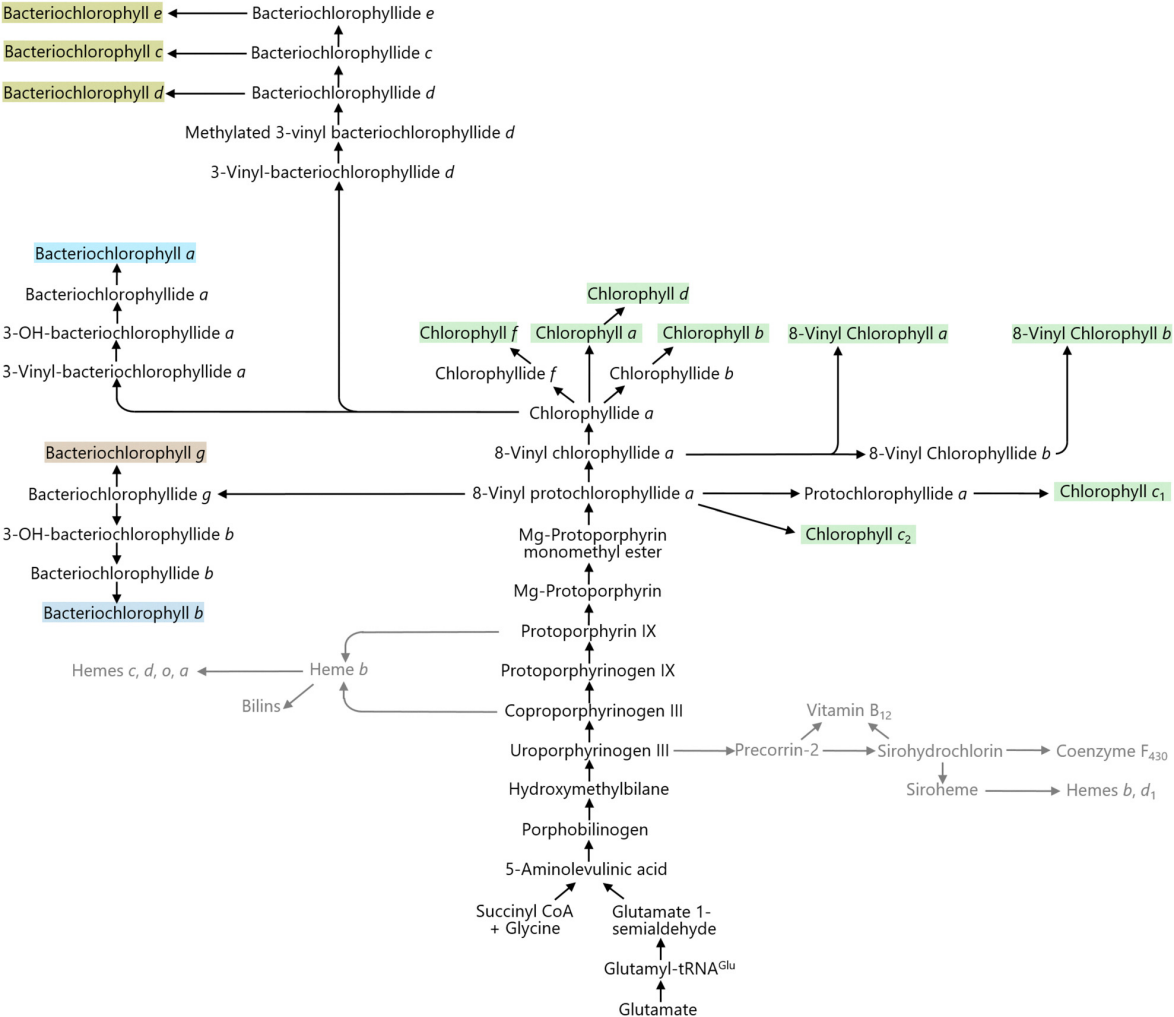
The biosynthetic pathways for Chls and BChls, including the routes (in grey) from uroporphyrinogen III leading to vitamin B_12_, siroheme, cofactor F_430_, heme *b* and heme *d*_1_. The steps from uroporphyrinogen III then lead to PPIX, the precursor of hemes *a*, *b*, *c*, *d* and *o*, and the bilins (also in grey). Insertion of Mg into the protoporphyrin macrocycle commits this part of tetrapyrrole metabolism to the biosynthesis of BChls and Chls.

The four-orbital model of Gouterman^17^ (also discussed in ref. 11, 18 and 19) can account for porphyrin absorption bands; in this model the two highest occupied molecular orbitals (HOMO) and two lowest unoccupied π-molecular orbitals (LUMO) result in four possible HOMO to LUMO π–π* transitions. Excited state orbitals are intermixed, but also polarised along the *X* and *Y* axes shown in Fig. 1 and 2, which run between the pyrrole nitrogen atoms on rings A and C (*Y*), and rings B and D (*X*). For a completely symmetrical conjugated system, the *X*- and *Y*-polarised transitions are degenerate and the main absorption feature for such a molecule is a strong B-band (Soret band) in the 380–420 nm region. In practice, the symmetry of porphyrin structures found in nature, such as PPIX in Fig. 3 and Chls *c*_1_, *c*_2_ and *c*_3_ in Fig. 2, is weakened, in the former case by the opposing central hydrogens on two of the four pyrrole rings. In the latter, insertion of Mg in the *c*-type Chls removes the asymmetry arising from the pyrrole hydrogens, but addition of the 13^1^-keto group conjugated to the macrocycle provides another source of asymmetry. The consequence in each case is some loss of degeneracy in the excited states associated with the *X* and *Y* transitions and a splitting of the absorption, yielding B (B_*X*_, B_*Y*_) bands in the UV/blue region of the spectrum and Q (Q_*X*_, Q_*Y*_) bands in the visible region.

#### 2.1.1. Chlorophylls *c*_1_, *c*_2_ and *c*_3_

**Fig. 4:**
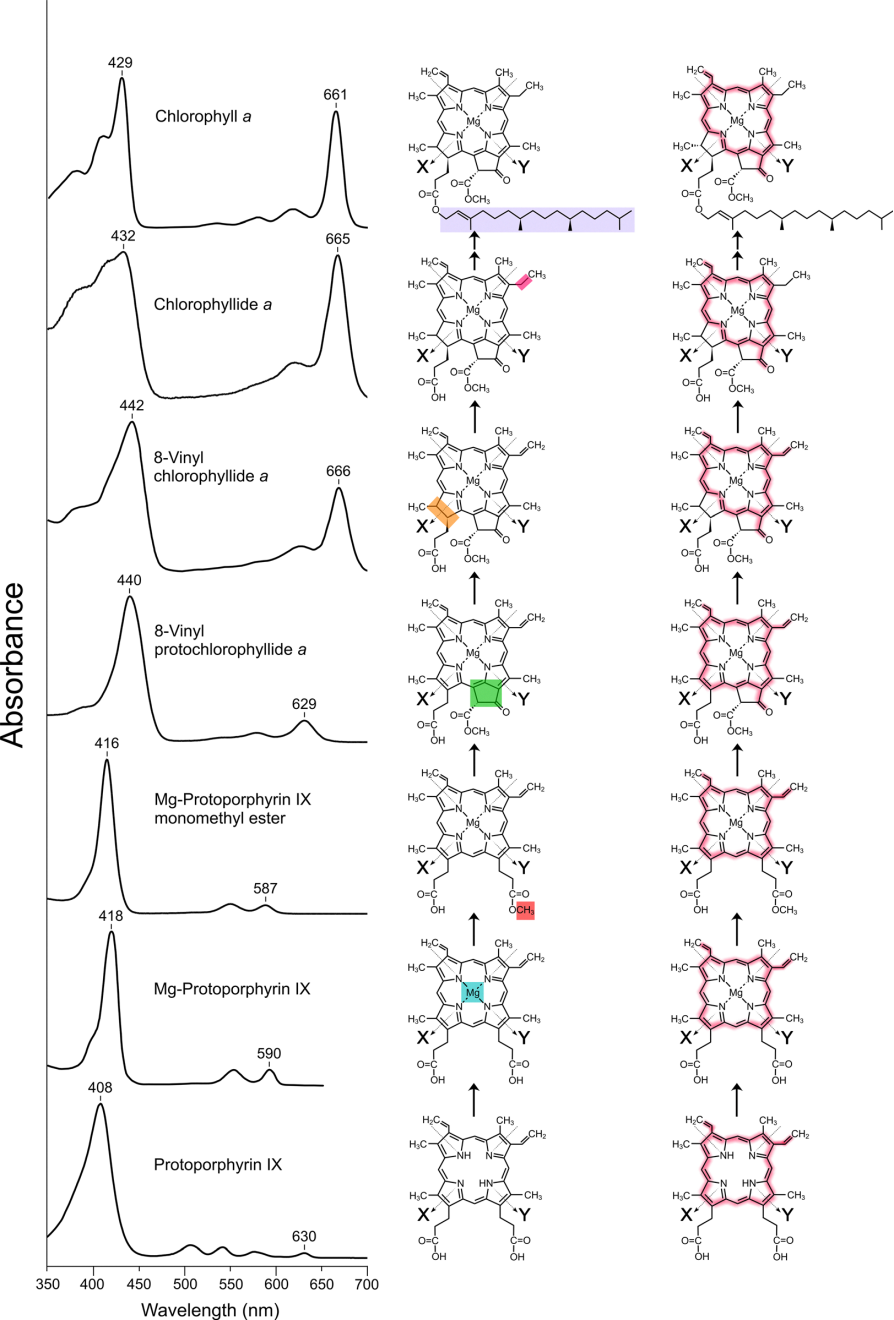
The biosynthetic pathway leading to Chl *a*, depicted in three ways. (Left) The progression towards Chl *a* visualised as a series of absorption spectra, for the pigments solvated in methanol (but with PPIX in chloroform), showing the wavelength positions of major absorption maxima. Two arrows represent the successive addition of GG, then reduction to phytyl; both pigments have the same absorption spectrum. (Middle) The chemical structures of the biosynthetic intermediates are shown, with coloured boxes used to indicate the various modifications, according to the colour scheme used in.^14^ (Right) The chemical structures of the biosynthetic intermediates, with diffuse red outlines used as in Fig. 2 to illustrate the successive changes in macrocycle conjugation, as the pathway progresses towards the final product, Chl *a*.

The *trans*-acrylate group at C17, which is unique to these pigments, extends macrocycle conjugation along the Q_*X*_ axis (Fig. 2). The absorption of Chl *c*_1_, with strong B-band absorption at 439 nm and weak absorption around 630 nm, closely resembles the absorption of the Chl biosynthesis intermediate 8-vinyl protochlorophyllide (8V-PChlide; Fig. 4), also called 3,8 divinyl protochlorophyllide (DV-PChlide). Further small B-band shifts are imparted by the 8V (Chl *c*_2_) and 7-methoxycarbonyl (Chl *c*_3_) groups. A full discussion of all aspects of Chls *c*_1_, c_2_ and *c*_3_ can be found in ref. 20, which details several more Chl *c* variants, as well as discussing their evolution, biosynthesis and function in the light-harvesting complexes of brown algae and diatoms. Fig. 2 shows that Chls *c*_1_, *c*_2_ and *c*_3_ have the highest B/Q band intensity ratio among all the Chls and BChls; the strong B-band absorption is functionally important because organisms with *c*-type Chls are particularly suited to harvest the blue light that selectively penetrates into deep water.^12,20,21^ Chls *c* perform an auxiliary role in light harvesting and they transfer absorbed energy to the more abundant Chl *a*.^20^

### 2.2. Chlorins

Ring D, through which the *X*-axis runs, is reduced in all these pigments. As a result of removing the C17–C18 π-bond, orbitals associated with excited states along the *Y* axis gain oscillator strength,^11^ redshifting and intensifying Q_*Y*_ absorption as seen with Chls *a*, *b*, *d* and *f* (Fig. 2). Thus, Q_*Y*_ absorption for Chl *a* at 661 nm is much stronger than the ∼630 nm Q_*Y*_ band for Chls *c*_1_, *c*_2_ and *c*_3_ (Fig. 2). The biosynthetic pathway depicted in Fig. 4 also illustrates the consequences reducing the C17=C18 double bond; the conversion of 8V-PChlide to 8V-chlorophyllide (8V-Chlide) redshifts the Q_*Y*_ band from 629 nm to 666 nm, while also increasing its amplitude. Relative to porphyrins, this basic pattern of reconfigured conjugation and redshifted, intensified Q_*Y*_ absorption is found in all the chlorin pigments, namely Chls *a*, *b*, *d* and *f* and BChls *c*, *d*, *e* and *f* (Fig. 2). Exocyclic conjugated double bonds impart further spectral changes, and account for differences between Chls *a*, *b*, *d* and *f*.

#### 2.2.1. Chlorophylls *a*, *b*, *d* and *f*

These are the most globally abundant pigments by virtue of Chl *a*, which is the main light absorber on Earth for oxygenic photosynthesis.^19^ The ubiquity and abundance of Chl *a* are apparent from satellite imaging, which shows how variations in temperature and nutrient availability affect the distribution of Chl *a* in the oceans as well as displaying the huge seasonal fluctuations in terrestrial Chl *a*.^22–24^ The absorption spectrum of Chl *a* (Fig. 2) leaves a ‘green gap’ between 450–600 nm, filled in photosynthetic organisms by a range of accessory light-absorbing pigments such as carotenoids and bilins, which transfer energy to the Q_*Y*_ absorption band of Chl *a*.

One such accessory pigment is Chl *b*, which is found in light-harvesting complexes of algae and plants.^25^ Unlike Chl *a*, Chl *b* has a 7-formyl group that affects conjugation along the *X*-axis, redshifting the B-band from 429 to 454 nm while also blueshifting and attenuating Q_*Y*_ absorption. These attributes narrow the ‘green gap’ (Fig. 2) and the complementary absorption properties of Chls *a* and *b*, together with carotenoids, underpin the essential role of Chl *a*/*b* (LHCII) complexes as the major light-harvesting complexes in plants and algae.^26–28^ Indeed, the LHCII complex is likely the most abundant membrane protein on Earth.^29^ Chls *a* and *b* are closely packed within the LHCII complex, and energy absorbed by the Chl *b* B band can transfer rapidly and efficiently to the Chl *a* Q_*Y*_, *via* the Chl *b* Q_*Y*_ band at 644 nm.^30^ LHCII donates harvested energy to Photosystem II (PSII) and Photosystem I (PSI) complexes,^26–28^ but it is more than just an absorber and transmitter of energy; LHCII can be considered as a ‘smart antenna’ that can regulate its light-harvesting function by dealing with large fluctuations in solar irradiance. Instead of feeding excitation energy to photosystem complexes LHCII can switch to a quenching mode, sparing its acceptor partners the damaging consequences of excessive energy input.^31^

The 8V derivatives of Chls *a* and *b* (also known as Chls *a*_2_ and *b*_2_) are not shown in Fig. 2, but the effect of converting the 8V group to an 8-ethyl (8E) group can be seen in the biosynthetic pathway (Fig. 4). The B-band absorption of 8V-Chlide *a* (often called DV-Chlide *a*) is redshifted and relatively stronger than for the monovinyl pigment (Chlide *a* in Fig. 4, but also called 3V, 8E-Chlide *a*, 3V-Chlide *a*, or MV-Chlide *a*). Similarly, the 8V group in 8V-Chl *a* shifts the B-band maximum from 429 nm in Chl *a* to 436 nm (for pigments in Et_2_O), with no significant effect on the position of the Q_*Y*_ band; there is also a small (∼7%) increase in the B-band amplitude relative to Q_*Y*_ (not shown). These small alterations in B-band absorption are important for some marine cyanobacteria, conferring marginal gains in their ability to harvest blue light, the only wavelengths that can penetrate down to 100 m or more in the oceans.^12,21^ This group of bacteria includes the globally abundant picocyanobacterium *Prochlorococcus*, so the 8V-Chls of *Prochlorococcus* represent a substantial proportion of the mass of marine Chls.^32–34^

Chl *d* provides another example of an exocyclic conjugated double bond affecting absorption; in this case the 3-vinyl group on ring A of Chl *a* is replaced with the more electron-withdrawing 3-formyl group. This change selectively modifies electron density along the *Y*-axis, increasing Q_*Y*_ intensity and redshifting the B and Q_*Y*_ bands relative to Chl *a* by 15 nm and 25 nm, respectively. Thus, the 3-formyl group enables *Acaryochloris marina*, a cyanobacterium that makes Chl *d*, to use light for photosynthesis that its Chl *a*-utilising neighbours cannot. *A. marina* was originally found in a biofilm that lies underneath the didemnid ascidian *Lissoclinum patella*,^35^ but it is now known to be widespread in environments enriched in far-red light.^36^

Chl *f* is the most redshifted of the Chl pigments, a consequence of being the only Chl with two exocyclic conjugated groups on ring A. The additive, electron withdrawing effects of the 2-formyl and 3-vinyl groups extend electron density along the Q_*Y*_ axis relative to Chls *a*, *b* and *d*, and continuing the trend (Chls *b*–*a*–*d*–*f*) of increasing Q_*Y*_ : B band intensities and redshifted Q_*Y*_ maxima (Fig. 2). Chl *f*, which was discovered only recently in 2010,^37^ has a Q_*Y*_ absorption maximum at 695 nm, and the assembly of this pigment within the photosystems of some cyanobacteria has allowed adaptation to life in spectrally filtered light environments, which are enhanced in far-red wavelengths above 700 nm.

#### 2.2.2. Bacteriochlorophylls *c*, *d*, *e* and *f*

These BChls form a distinct grouping, with modifications at C3^1^, C8^2^, C12^1^, C13^2^, and C20. Another distinguishing feature is C17^3^ esterification with farnesyl rather than the longer phytyl chain found in most of the Chls and BChls. In the green filamentous bacterium *Chloroflexus aurantiacus* C17^3^ is primarily esterified with stearyl alcohol.^38^ These are the only (B)Chls with a 3^1^-hydroxyl group, with a range of C8^2^ and C12^1^ substituents or with one at C20, and the only (B)Chls with no 13^2^-methoxycarbonyl group. Despite this range of distinctive modifications, they have minimal effects on the absorption spectra of BChls *c*, *d*, *e* and *f* in solvent, and their ∼420–460 nm B bands and 634–661 nm Q_*Y*_ bands have similarities with Chls *a* and *b* (Fig. 2). The C7 formyls in BChls *e* and *f* relative to BChls *c* and *d* have analogous effects to the C7 formyl of Chl *b* in relation to Chl *a*; altered conjugation along the *X*-axis redshifts the B-band, and blue-shifts and attenuates Q_*Y*_ absorption. It should be noted here that BChl *f* is not found in nature, and it is synthesised by a genetically engineered strain of the photosynthetic bacterium *Chlorobaculum* (*Cba*.) *limnaeum*.^39^

The modifications seen in BChls *c*, *d* and *e* reflect their deployment in unique light-harvesting arrays; all the other Chls and BChls in Fig. 2 rely on binding to protein scaffolds in order to function, whereas BChls *c*, *d* and *e* can self-assemble to form tightly packed, nanotubular, supramolecular structures, enclosed by a thin, protein-stabilized glycolipid membrane. Up to 250 000 of these BChls form ovoid assemblies called chlorosomes, which in bacteria such as *Cba. tepidum* can be as large as 133 × 57 × 36 nm.^40,41^

Chlorosomes can vary in size and shape, and their overall absorption and fluorescence properties depend on whether they are assembled from BChls *c*, *d*, or *e*, but in general the C3^1^, C8^2^, C12^1^, C13^2^, C17^3^ and C20 modifications promote the serial, self-organising stacking of the macrocycles. The requirements for stacking BChl macrocycles *in vitro* have been extensively researched by the Tamiaki group and are now well understood.^42^ The 3^1^-hydroxyl is important because it ligates the central Mg atom of an adjacent BChl, so the stacking process is cooperatively extended and enhanced.^40^ The other modifications, such as the absence of the 13^2^-methoxycarbonyl group and methylation at C8 and C12, affect the packing between adjacent BChls. For example, methylation of the C8 ethyl to produce propyl, isobutyl, and neopentyl side chains, and methylation of the C12 methyl to produce an ethyl side chain, are used to fine-tune packing of the BChl macrocycles, thereby modulating the absorption of the chlorosomes.^40,43^ As a result of forming extended, excitonically coupled arrays, the absorption of chlorosomes is greatly redshifted relative to the spectra in Fig. 2 so BChl *c*, with a Q_*Y*_ absorption maximum of 661 nm, is shifted as far as 750 nm in *Cba. tepidum.*^43^ Thus, the chemical organisation of this group of BChls determine macroscopic organisation and light-harvesting function.

### 2.3. Bacteriochlorins

Large bathochromic shifts are also found for protein-bound assemblies of BChls *a*, *b* and *g*. This small group of pigments exhibits the most redshifted Q_*Y*_ absorption maxima (Fig. 2), which arises from the combined effects of reducing the C7=C8 and C17=C18 double bonds. The lower degeneracy of excited states associated with the *X* and *Y* transitions leads to further splitting of B and Q absorption, with B_*X*_, and B_*Y*_ bands in the UV/blue region 30–40 nm apart and Q_*X*_ and Q_*Y*_ bands separated by as much as 170 nm in the case of BChl *b*. For comparison, the Q_*X*_, and Q_*Y*_ bands for Chl *a* are only 35 nm apart (Fig. 2). Compared to the chlorins, reduction of the opposing rings B and D further limits the extent of macrocycle conjugation along the *X* axis, and Q_*Y*_ absorption for BChls *a*, *b* and *g* shifts even more to the red, almost to 800 nm. Thus, photosynthetic organisms with BChls *a*, *b*, or *g* as antenna and RC pigments can occupy spectral niches that are inaccessible to Chl-producers.^21^

## 3. An overview of the chlorophyll and bacteriochlorophyll biosynthesis pathways

This perspective will focus on the biosynthetic pathway for Chl *a*, given its importance as the main light-absorbing pigment on Earth. The seven biosynthetic reactions from PPIX to Chl *a* are just a small subset of the metabolic network that synthesises the tetrapyrrole cofactors required for respiration, nitrogen and sulfur metabolism, photosynthesis, mammalian metabolism, and methanogenesis.^44^ The ∼90 interrelated reactions that form these ‘molecules of life’, namely hemes, bilins, Chls, vitamin B_12_ and the F_430_ cofactor, have been compiled into an interlinked tetrapyrrole roadmap,^44^ a simplified version of which is depicted in Fig. 3. The central spine of the Chl/BChl pathways leads from 5-aminolevulinic acid to 8V-PChlide *a*, *via* a series of intermediates with central importance to the rest of the tetrapyrrole network. Thus, vitamin B_12_, cofactor F_430_, siroheme and hemes are derived from uroporphyrinogen III, and PPIX is the precursor of hemes *a*, *b*, *c*, *d*, *o*, and the bilins (Fig. 3). Importantly, PPIX also acts as the substrate for the Mg chelatase (MgCh) enzyme complex that catalyzes insertion of Mg into the protoporphyrin macrocycle, committing this part of tetrapyrrole metabolism to the biosynthesis of (B)Chls. The BChl/Chl biosynthetic steps then proceed until the formation of 8V-PChlide *a*, the last common intermediate for the family of BChls and Chls. Here, 8V-PChlide *a* can form the substrate for synthesising BChls *b* and *g*, or Chls *c*_1_ and *c*_2_ (Fig. 3), or it can be converted into 8V-Chlide *a*, which can be considered as a hub for biosynthesis of nearly all Chls and BChls.^44^ One set of reactions from 8V-Chlide *a* yields the 8V versions of Chls *a* and *b*, which play an important role as the major pigments in the globally abundant picocyanobacterium *Prochlorococcus*.^32–34^ Continuation of the main biosynthetic route leads next to Chlide *a*, from which most Chls and BChls originate. Thus, Chls *a*, *b*, *d* and *f*, as well as BChls *a*, *c*, *d* and *e*, are formed from Chlide *a* (Fig. 3).

### 3.1. The structures and absorption properties of biosynthetic intermediates leading to chlorophyll *a*

The biosynthetic steps outlined in Fig. 4 introduce a series of structural and energetic properties that enable Chl *a* to participate not only in absorbing solar energy in antenna complexes, but also in converting and transiently storing this energy in the form of a charge-separated state in RC complexes.^19^ These steps are mediated by a series of enzyme-catalysed reactions that progressively strengthen and redshift the absorption band associated with the Q_*Y*_ transition dipole.

At the start of the pathway, PPIX has strong B-band absorption in the 380–420 nm region, but only minimal Q_*Y*_ absorption between 590–630 nm. Then, Mg is inserted, with only small effects on absorption, but with pervasive and powerful effects on function. Several metals, Fe for example, could also confer the ability to form ligands on protein side chains, but nature has selected Mg as the central metal. Mg has a coordination number of six, strongly preferring oxygen-containing ligands such as water, but also able to form stable ligands to the pyrrole nitrogen atoms of Chls (and their biosynthetic intermediates), as well as to protein side chains such as histidine residues. However, in most cases the central Mg of Chls and BChls in photosynthetic complexes is pentacoordinated.^45^ Thus, single or paired Chls, ligated to proteins, are held in place for energy transfer or photochemical functions, while also contributing decisively to the overall stability of Chl–protein complexes. Similarly, stacked BChls *c*, *d*, or *e* in chlorosomes are stabilised in part by their 3^1^-hydroxyls ligating to the central Mg of an adjacent BChl.^40^ Mg in chlorins and bacteriochlorins also maximises the excited state lifetimes of the pigments, which enables participation in energy transfer and redox reactions, while minimising formation of potential toxic triplet states due to intersystem crossing from the singlet state.^9,19^ The strong ligands formed by the central Mg within protein binding sites allow proteins to exert control over the aggregation state and electrostatic environment of Chls and BChls, contributing to their remarkable and contrasting ability within RCs to generate either strongly oxidising or reducing Chl species.^19^

The next pigment in the pathway, Mg-PPIX monomethyl ester (MgPME), is also very similar in absorption to the preceding intermediates (Fig. 4), but addition of a methyl group to the C13 propionic acid group is a prerequisite for the subsequent and crucial formation of the isocyclic ring E.^11^ This fifth ring is found in all Chls and BChls, so it imparts essential structural and functional properties. The presence of the isocyclic pentanone ring E braces the macrocycle and as a result the C=O group at C-13^1^ of ring E is coplanar with the rest of the macrocycle,^9^ which promotes delocalisation of the conjugated π-system along the Q_*Y*_ axis and redshifting of absorption from 587 nm to 629 nm (Fig. 4). Lowered macrocycle flexibility due to ring E is suggested to help prevent dissipation of excited states due to internal conversion.^9^ Another functional aspect of the 13^1^-keto group is forming important hydrogen bonding interactions within antenna complexes, for example.^46,47^ The other major peripheral group on ring E is the methoxycarbonyl at C13^2^, which is one of the three stereochemical locations in Chl *a* (aside from those in the phytyl chain), the others being at C17 and C18, and with *R*, *S*, *S* configurations, respectively.^10,11^ Enolisation then reprotonation of Chl *a* at C13^2^ forms the *S*-stereoisomer, termed Chl *a*′,^9,10,48^ which forms part of the charge-separating core of Type 1 RCs.^49^ A pair of BChl *g*′ epimers forms the primary electron donor in RCs of the anoxygenic phototroph *Heliomicrobium modesticaldum*.^50,51^ The C13^2^ methoxycarbonyl is sterically significant and although it is an invariant feature of all Chls and the ‘true’ BChls *a*, *b* and *g* (Fig. 2), it is absent from BChls *c*, *d* and *e*, where it would otherwise impede the serial stacking of the macrocycles within chlorosomes.^40,43^

The next biosynthetic step forms a chlorin, and reduction of ring D at C17–C18 provokes a major absorption change, as outlined earlier, because of the altered macrocycle conjugation (Fig. 4). The balance between the oscillator strengths associated with the Q_*X*_ and Q_*Y*_ axes changes, increasing and redshifting Q_*Y*_ absorption from 629 nm to 666 nm, which is more or less the final absorption position for Chl *a* (see Fig. 4). Thus, the final three biosynthetic steps do not exert much effect on the wavelength maximum of Q_*Y*_ absorption, although reduction of the 8V group to 8E blue-shifts the B-band from 442 nm to 432 nm. As mentioned earlier, oceanic bacteria such as *Prochlorococcus* benefit from retaining the vinyl at C8 and the attendant 442 nm absorption, because only blue light is available at depths of 100 m or more. Conversely, a C8 ethyl group increases the amplitude of Q_*Y*_ absorption relative to the B-band maximum by ∼60% (Fig. 4), which allows cyanobacteria growing in shallow water, or terrestrial photosynthesisers, to absorb more red light.

The next biosynthetic step esterifies the C17 propionate group with a long-chain alcohol, usually geranylgeraniol, which is followed by the stepwise reduction of geranylgeranyl Chl (Chl_GG_) to phytyl Chl, *via* dihydrogeranylgeranyl Chl (Chl_DHGG_) and tetrahydrogeranylgeranyl Chl (Chl_THGG_). In principle, the esterification and reduction steps could proceed in a different order, with prior reduction of geranylgeranyl pyrophosphate (GGPP) to phytyl PP (PPP), followed by its attachment to Chlide *a* forming Chl *a* (phytyl Chl *a*). Although the final esterifying moiety is generally phytyl, there are examples where GG is found, in the case of BChl *a* in *R. rubrum*,^52^ and farnesyl in the case of BChls *c*, *d*, *e*, *f* and *g*.^40^ The green bacteria that synthesise BChls *c*, *d*, *e* and *f*, can esterify the C17 propionate with geranylgeraniol, phytol, 2,6-phytadienol, hexadecanol and octadecanol.^11,44^

The absorption and excited state properties of the pigments in solvent are not affected significantly by C17^3^ esterification, but the addition of the long-chain (C_20_H_40_O) isoprenoid phytol to monovinyl Chlide *a*, for example, represents a major modification that accounts for a third of the molecular mass of Chl *a*, while also increasing its hydrophobicity with important consequences for membrane location, the packing of Chls, and their attachment to proteins. The addition of phytol is a therefore decisive and essential step in photosystem assembly.^53–55^ The disposition of the phytyl tails has been revealed by numerous high-resolution structures of photosynthetic complexes, which show that the phytyls influence pigment–pigment and pigment–protein distances and orientations, sometimes intertwining or associating with carotenoids (for example^45,56,57^).

## 4. The enzymes of chlorophyll *a* biosynthesis

The following sections outline the enzymes that catalyse the biosynthesis of Chls *a*, *b*, *c* and *f*, but more depth and detail covering historical, regulatory and physiological aspects can be found in reviews such as,^6–11,13,20,58^ and we do not cover the steps unique to the BChl pathways. Indeed, each Chl biosynthesis step merits its own review, but here the focus is on the structures and mechanisms of the Chl pathway enzymes. We have used AlphaFold 3 (AF3)^59^ to augment the existing experimentally determined structures, permitting a complete structural overview of the Chl biosynthesis enzymes. The performance of AF2 and AF3 has been tested previously using critical assessment of structure prediction (CASP),^60,61^ and their value demonstrated by providing starting models for X-ray crystallographic and cryogenic electron microscopy (cryo-EM) model reconstruction,^62–65^ and in the design of structurally validated *de novo*-designed proteins.^66,67^ A set of useful parameters has been compiled for the modelling of Chl pathway enzymes, and can be found in Table S1 in supplementary information (SI). This includes the ipTM (interface predicted template modelling) confidence scores for all AF3 models and, where appropriate, the root mean square deviation (RMSD) values for AF3 *vs.* experimentally determined structures in the RCSB PDB (research collaboratory for structural bioinformatics protein data base, https://www.rcsb.org/). The zipped structure files for all models are also available at https://doi.org/10.1039/d6cb00082g.

### 4.1. Step 1 – magnesium chelatase

#### 4.1.1. Insertion of magnesium into porphyrins

The chemical mechanisms of metalation of porphyrins have been studied for decades (reviewed in ref. 68), and several steps have been identified, including deformation of the porphyrin ring, association of the solvated outer-sphere of the metal ion and the porphyrin, then exchange of an (unprotonated) porphyrin nitrogen atom with a solvent molecule in the coordination sphere. Further desolvation of the metal ion allows formation of the second metal–porphyrin bond, with the metal ion now coordinating both of the unprotonated pyrrole nitrogen atoms. The presence of hydrogen atoms on the other two pyrrole nitrogen atoms obliges the bound metal to sit above the porphyrin plane, forming a sitting atop (SAT) complex, as originally proposed by Fleischer and Wang.^69^ The rate of insertion of Mg into porphyrins follows the order Cu > Zn > Mn, Co, Fe > Ni > Cd > >Mg.^70,71^ Relative to other metals, Mg^2+^ has a lower affinity for porphyrin and a stronger affinity for water, so the six water molecules in the first coordination sphere are strongly polarised, creating a second hydration shell. Valuable insights into Mg insertion have been provided by density functional calculations, which followed the stepwise displacement of water molecules from the first hydration shell through to Mg–porphyrin formation *via* Mg–porphyrin SAT complexes.^72,73^ A ten-step reaction mechanism was proposed, starting with formation of an outer-sphere complex between the hydrated metal and the porphyrin, followed by exchange of one water ligand leading to formation of the first bond from Mg to a central pyrrole nitrogen atom. After two more such exchanges the second Mg–pyrrole N bond is formed, then the fourth and fifth water molecules are moved, modelled as transferring to the second coordination sphere,^73^ but this outer sphere might not be present in an enzyme-catalysed mechanism. At this stage there are four Mg–N bonds, with two of the pyrrole nitrogens still protonated, and with Mg retaining one of its original water ligands. Increasing the number of metal–porphyrin bonds is accompanied by progressive distortion of the porphyrin, with the mounting strain released when the two pyrrole NH groups are deprotonated and the metal moves from its SAT position into the ring plane.^73^

These theoretical studies highlight the problems that confront a Mg-chelating enzyme, which must remove five water molecules from the strongly bound hexacoordinate hydration shell and deprotonate two pyrrole nitrogens. Thus, the enzyme-catalysed insertion of Mg^2+^ into the PPIX macrocycle is energetically demanding; this requirement, and the need to regulate this first step in Chl biosynthesis, necessitate an ATP-fuelled, multi-subunit, allosterically controlled enzyme. Early experiments with recombinant proteins produced in *Escherichia coli* using *bchH*, *I* and *D* genes from *Rhodobacter* (*Rba*.) *sphaeroides*, or with *chlH*, *I*, and *D* genes from *Synechocystis* sp. PCC 6803 (hereafter *Synechocystis*), showed that magnesium chelatase (MgCh) is a three-subunit complex that catalyses the ATP-dependent conversion of PPIX to Mg-protoporphyrin IX (MgPIX).^74,75^ The ATP-driven catalytic cycle involves the ChlI (∼35 kDa) and ChlD (∼75 kDa) subunits, which are members of the AAA^+^ (ATPases associated with various cellular activities) superfamily.^76–79^ As many as 14 ATP are required to drive each catalytic cycle.^80^ ChlH (∼150 kDa) binds the PPIX substrate.^74,75,81^

#### 4.1.2. Structural, kinetic and mutagenesis studies of magnesium chelatase

Although there are currently no structures of the MgCh complex, early crystallographic work determined the structure of BchI from *Rba. capsulatus* and showed that it could form hexamers,^82^ subsequently also investigated by cryo-EM.^83,84^ A 2.9 Å resolution crystallographic structure showed that ChlI purified from *Synechocystis* forms hexamers,^85^ while a more recent cryo-EM study of ChlI from the nitrogen-fixing cyanobacterium *Nostoc* sp. PCC 7120 revealed pentamers and hexamers.^86^ An earlier, low resolution study revealed heptamers of BchI,^79^ so it is likely that the oligomerisation of I subunits is poorly controlled in the absence of the D subunit. The cryo-EM resolutions for the hexamers (3.8 Å and 4.0 Å) and for the pentamer (4.9 Å) were sufficient to show five ATP molecules and one ADP bound in hexamer conformation A, with four ATPs and two ADPs in conformation B. ATP hydrolysis was proposed to rearrange the hexamer ring, providing the basis for a motor function that couples ATP hydrolysis to Mg insertion.^86^

The 2.5 Å resolution crystal structure of ChlH from *Synechocystis* showed the overall architecture of this subunit and that the active site is buried within the protein interior.^87^ Subsequently, a combination of X-ray crystallography, computational modelling, mutagenesis and enzymology was used to identify the PPIX binding site, which is close to the catalytically essential E660 residue.^88^ The active site of ChlH is connected to the exterior by a solvent-filled channel, and the strictly conserved residue E625 sits at the interface with bulk solvent. E625 was suggested to play a role in delivering Mg to the active site, but this channel could also provide a route for abstracting protons from pyrrole N atoms during the insertion of Mg into PPIX.^88^

Unravelling the roles of ChlD is key for understanding MgCh, and the functionally crucial interaction between ChlD and ChlH (*K*_d_ ∼ 330 nM) was mapped using chemical cross-linking coupled with mass spectrometry, microscale thermophoresis, and by modifications that either truncate ChlD or modify single residues.^89^ The C-terminal integrin I domain of ChlD has a metal ion-dependent adhesion site (MIDAS) motif^90^ that mediates the Mg^2+^-dependent ChlD–H interaction. Five Glu residues in the C-terminal domain of *Synechocystis* ChlD, E510, E513, E600, E603, and E605, are important for the cooperative response of MgCh to Mg^2+^.^91^ ChlD binds *via* the integrin I domain to the body region of ChlH,^89^ and the AAA^+^ N-terminal domain of ChlD, which shares ∼40% sequence identity with ChlI, binds tightly to ChlI with a *K*_d_ ≈ 7 nM,^52,92^ which would close an arc of five ChlI subunits^86^ to form a six-membered I_5_D ring.

A series of kinetic studies on the cyanobacterial MgCh formed from recombinant enzyme subunits^79,80,91,93–96^ suggests a model where a heterogeneous ChlI_5_D ring interacts with the body region of the ChlH protein *via* the C-terminal integrin domain of ChlD.^89^ Hydrolysis of ATP by ChlI^79,80^ drives a conformational change transmitted initially to ChlD then to the ChlH–porphyrin complex, promoting the insertion of the Mg^2+^ ion into the PPIX ring.^88^ Thus, MgCh is a molecular machine that chemomechanically couples the free energy from ATP hydrolysis by the curved array of AAA^+^ ChlI subunits to the metal ion insertion site on ChlH, through the bridging subunit, ChlD.

#### 4.1.3. Computational modelling of the magnesium chelatase complex

**Fig. 5:**
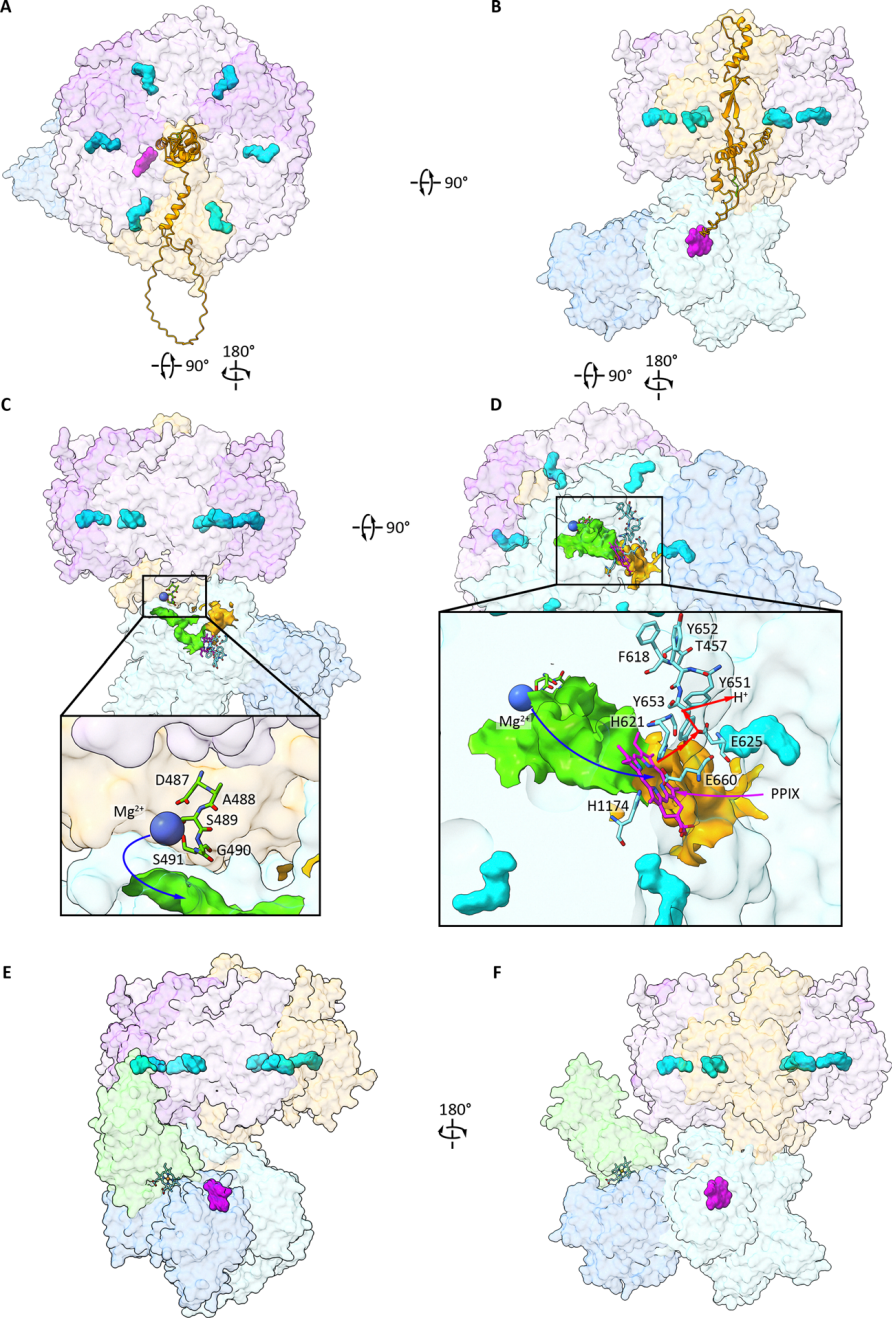
AF3 model of a putative magnesium chelatase complex highlighting the ChlD (pastel orange), ChlI (light mauve and light lavender) and ChlH (light blue – body; baby blue – head) subunits with the stoichiometry ChlHI_5_D. The PPIX substrate (magenta) sits in the body of ChlH and the ATP cofactors (cyan) bind at the ChlI–ChlI and ChlI–ChlD interfaces within the HI_5_D AAA^+^ complex. (A) and (B) Two surface views of the overall structure of the complex. The intrinsically disordered portion of ChlD (pastel orange ribbon) connects the ChlI-like portion of ChlD to the integrin, ChlH-interacting portion *via* the energy transducing subdomain (shown in cartoon representation) that sits within the AAA^+^ pore of the ChlI_5_D ring. (C) and (D) Insets showing detail of the putative Mg^2+^ (bright green) and PPIX substrate (orange) pores along with a Mg^2+^ ion (blue sphere) bound to the MIDAS motif at the entry to the putative Mg^2+^ channel. The AF3 modelled substrate, PPIX, is shown in stick representation (heteroatom colours: blue – nitrogen, red – oxygen, magenta – carbon) at the confluence of the PPIX and Mg^2+^ channels. Previously identified essential catalytic residues H1174, E660 and H621 (cyan, stick representation) point directly to the center of Mg^2+^-chelating tetrapyrrole nitrogen atoms. Blue and magenta arrows indicate the direction of substrate entry to the active site for Mg^2+^ and PPIX respectively, along with a putative proton conductance channel that proceeds along H621, Y653, E625 and Y651, highlighted with a red arrow. (E) and (F) Model of the MgCh–Gun4 complex with Gun4 (pastel green) sitting on the head group of ChlH, where the Gun4 tetrapyrrole binding site forms part of the binding interface. See Table S1 in the SI for ipTM scores for AF3 models and RMSD values for the correspondence of AF3 models with experimentally determined structures in the RCSB PDB.

A structure of the three-subunit MgCh enzyme would reveal essential mechanistic information, so new computational tools have been used to build a model of this complex, shown in Fig. 5. AF3 was used to calculate a hypothetical structure of the ChlHI_5_D complex. The model incorporates existing data for the ChlD–H interaction^89^ and for PPIX binding within ChlH.^88^ The HI_5_D stoichiometry in the model in Fig. 5 is consistent with native mass spectrometry analyses, and migration on native gel electrophoresis (P. J. Jackson and A. A. Brindley, personal communication). This model provides only a single snapshot of the complex, but the central position of ChlD shows how it might transmit free energy from ATP hydrolysis by the ring of ChlI subunits to ChlH, where it is used for metal ion insertion.

The crystallographic and cryo-EM structures of rings formed by the AAA^+^ protein ChlI show a central pore,^85,86^ which is a common feature of other AAA^+^ complexes.^97,98^ The AAA^+^ family uses the free energy from hydrolysing ATP to perform mechanical work, acting as molecular screws, unwinders, or threaders of a substrate within this central cavity.^98^ The model in Fig. 5 depicts a heterogeneous I_5_D ring, where the N-terminal half of D, which has homology with ChlI, replaces a 6th I subunit while the central pore encloses D-subunit residues 413–478. This extended segment of ChlD forms a right-handed pseudo-helical fold, which was predicted independently by AF3 and also ESMFold;^99^ near to the point of entry into the I_5_D pore D413–D459 wrap around a central core formed by residues D459–D478. The end of this central core directly connects to a beta-sheet (D479–D487), at the end of which is the MIDAS motif (D487–D491).

By analogy with other AAA^+^ complexes, and in line with the molecular machine concept, hydrolysis of ATP by the ChlI components performs mechanical work that is transmitted to a spindle formed by the ring-enclosed residues D413–D478 (coloured in orange in Fig. 5A and B), which represent the AAA^+^ substrate. In our model, chemomechanical forces are transmitted sequentially from the ChlI_5_D ring to residues D459–D478, subsequently to residues D413–D459, then to the MIDAS motif (D487–491). Extrusion of the MIDAS-bound Mg^2+^ ion along the pore in ChlH (blue arrow in Fig. 5C; pore coloured green), is proposed to be accompanied by progressive removal of at least some waters from the hydration shell of Mg^2+^, which enters the active site within ChlH. PPIX also enters this site (Fig. 5D inset, magenta arrow) *via* another channel (Fig. 5D inset; shown in orange). Large patches of basic residues (not shown) in ChlH regions 105–184 (body domain) and 852–888 (head domain), are proposed to associate with the outer leaflet of the thylakoid membrane. The acquisition of PPIX is likely to take place near to or at the membrane surface, and the initial PPIX binding site environment must change to become an enclosed chamber where no external waters are admitted, likely requiring large domain movements in ChlH. The chemistry of Mg^2+^ insertion into PPIX within the active is likely to follow some of steps identified by density functional calculations.^72,73^ The red arrow (Fig. 5D, inset), indicates a possible pathway for conducting protons removed from the central pyrrole nitrogen atoms of PPIX, which is part of the chelation process (see earlier section). All of these events, from movement of PPIX and the initially hydrated Mg^2+^ into the catalytic site, the insertion of Mg^2+^ into the porphyrin macrocycle and deprotonation of the pyrrole nitrogens, then product release, are all somehow driven by conformational changes in ChlH fuelled by ATP hydrolysis, and these processes need to be resolved by structures of catalytic intermediates in the coming years.

#### 4.1.4. Structure and function of the Gun4 subunit

The multi-subunit nature of MgCh, and its slow, energetically costly catalytic cycle,^80,93,95^ sharply contrasts with ferrochelatase (FeCh), which is a single polypeptide of ∼40 kDa that catalyses the rapid and energetically favourable insertion of Fe into PPIX.^73^ The Fe and Mg chelation steps launch porphyrins down the heme and Chl branches of tetrapyrrole metabolism, respectively (Fig. 3), so it is strategically and physiologically important to regulate this branchpoint, while taking into account the widely differing catalytic capacities of the FeCh and MgCh enzymes. Allocating the correct levels of flux down these branches must also respond to environmental variations such as changes in light and temperature, matching the supply of Chls with the assembly and repair of photosystems while avoiding the accumulation of potentially phototoxic Chl intermediates. ChlD is the regulatory hub for the MgCh enzyme complex,^89,94^ and the AAA^+^ site in ChlD is involved in the allosteric and cooperative responses of MgCh to both Mg^2+^ and MgATP^2^;^94^ it has been shown that ChlI2 of *Chlamydomonas reinhardtii* can stimulate chelatase activity by phosphorylating the integrin-I domain of ChlD.^100^ The Mg-dependent binding between ChlD and ChlH, involving the C-terminal integrin I domain of ChlD, mediates the cooperative response of the *Synechocystis* chelatase to Mg.^89,91^

Another important aspect of regulating MgCh involves the auxiliary porphyrin-binding subunit, Gun4.^101^ High-resolution structures of this small, soluble, 25 kDa protein from cyanobacterial sources revealed non-conserved N-terminal and conserved Gun4 domains linked by a 12–15 residue loop.^102,103^ Extensive kinetic analyses showed that Gun4 dramatically enhances the sensitivity of MgCh to Mg^2+^, so although there is almost no activity at 2 mM Mg^2+^ the complex is fully active in the presence of Gun4.^102^ The *Oryza sativa* GUN4 increases the maximum reaction rate of Mg chelation 16-fold;^104^ lower Chl levels, PPIX accumulation, and lowered MgCh and FeCh activities were found in *gun4* mutants of *Synechocystis*.^105,106^ A subsequent crystallographic study of Gun4-porphyrin complexes revealed ‘half-open’ binding sites for deuteroporphyrin IX (DIX; a more water-soluble analogue of PPIX) or magnesium deuteroporphyrin IX (MgDIX), compatible with a role for Gun4 in transferring porphyrins to biosynthetic enzymes.^107^ Gun4 from the green alga *C. reinhardtii* binds bilins,^108^ further indicating its role as a regulator of tetrapyrrole biosynthesis.^109^

The panels in Fig. 5E and F show a computational model in which the tetrapyrrole-binding face of Gun4 (heme coloured in green) binds to the head domain of ChlH. This binding site could be partially mediated by a tetrapyrrole, consistent with a regulatory role for Gun4 that could involve responding to flux down the heme/bilin and Chl branches of tetrapyrrole metabolism.

### 4.2. Step 2 – magnesium-protoporphyrin IX O-methyltransferase

The second step in Chl biosynthesis involves addition of a methyl group from *S*-adenosyl-l-methionine (SAM) to the C13 propionate of MgPIX, catalysed by Mg–protoporphyrin IX O-methyltransferase. This modification prepares the porphyrin for the next reaction, which forms the isocyclic ring E.^11^ Early steady-state kinetic studies on recombinant ChlM from *Synechocystis* using the MgDIX substrate showed that MgDIX and SAM bind to ChlM *via* a random ternary mechanism, with parameters *K*^SAM^_M_ = 38 µM, *K*^MgDIX^_M_ = 2.37 µM, *k*_cat_/*K*^SAM^_M_ = 1500 M^−1^s^−1^.^110^ A subsequent transient kinetics study showed that rapid binding of MgDIX to ChlM (>600 s^−1^) is followed by a slower (70 s^−1^) isomerization of the enzyme.^111^ Pre-steady-state catalysis, monitored using quenched-flow and high-performance liquid chromatography, showed evolution of a catalytic intermediate (rate constant of 11.9 ± 0.5 s^−1^), the decay of which (11.8 ± 0.5 s^−1^) coincides with the evolution of the Mg–deuteroporphyrin IX monomethylester (MgDME) product; given that *k*_cat_ is 0.057 s^−1^ release of the MgDME and S-adenosylhomocysteine (SAH) products is likely rate-limiting under the conditions of this assay.^111^

**Fig. 6:**
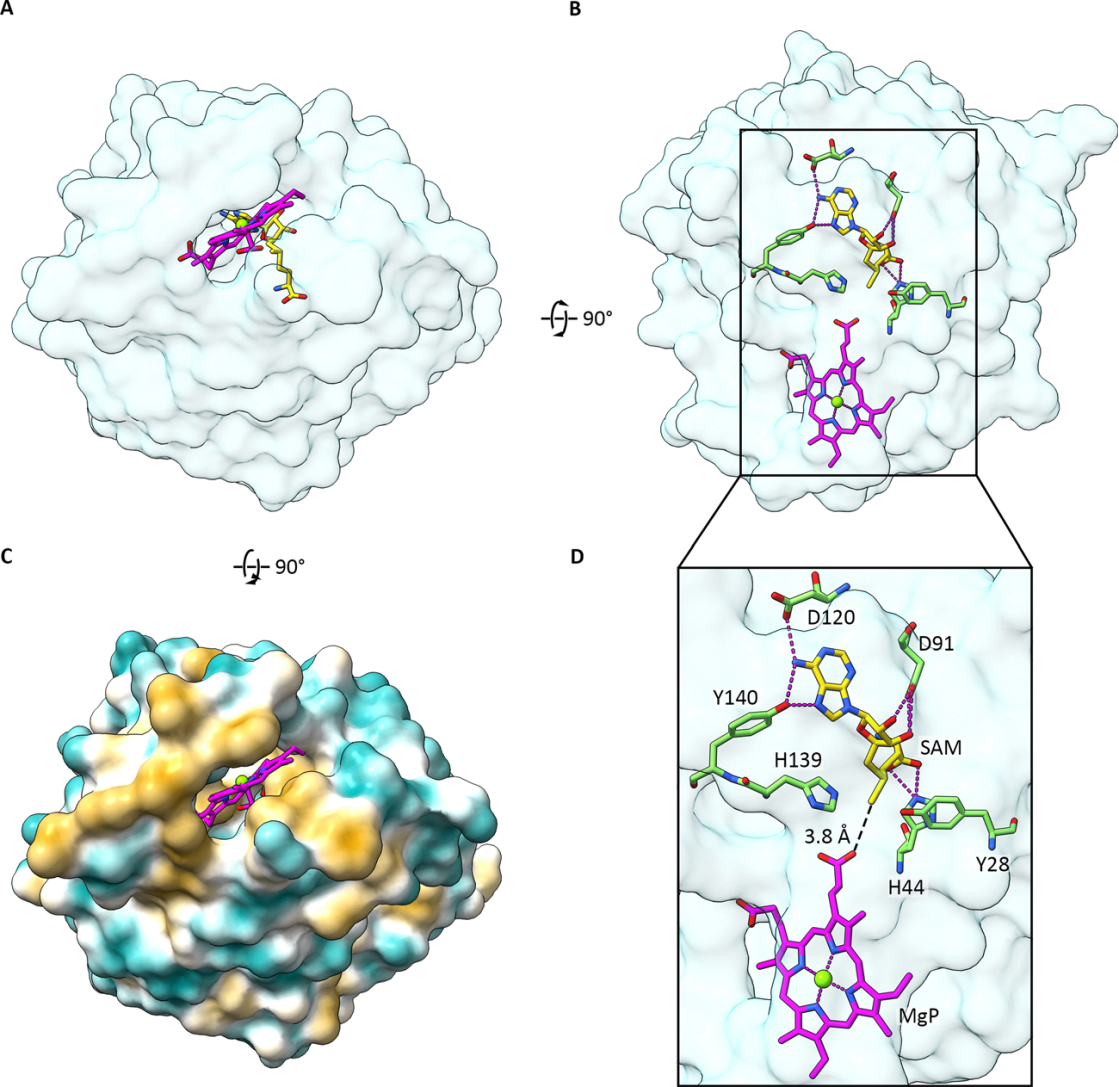
AF3 model of Mg-Protoporphyrin IX O-methyltransferase. (A) and (B) Two surface views of the overall topology of the enzyme with MgPIX and SAM shown in magenta and yellow, respectively (heteroatom colours: blue – nitrogen, red – oxygen). (C) View of the open substrate binding cleft of the enzyme highlighting the surface hydrophobicity (gold – hydrophobic, blue – hydrophilic) of the protein and indicating the potential for this face of the enzyme to dock onto the photosynthetic membrane where the substrate resides. (D) Enlarged view of the box in (B) highlighting important residues involved in SAM binding – the H44 pyrrole nitrogen interacts with the carboxyl group of the SAM methionine, the D91 carboxyl group interacts with the alcohol groups of the ribose moiety, and the Y140 hydroxyl and D120 carboxyl groups interact with the adenine moiety of SAM. Y28 and H139 are highlighted due to their importance for catalysis and are found either side of the axis running from the MgPIX propionate group to the methyl group of SAM. Aside from a hydrophobic cleft, no obvious interacting groups were found for the MgPIX substrate. The distance from the SAM methyl group to the substrate propionate carboxyl was modelled to be 3.8 Å. See Table S1 in the SI for ipTM scores for the AF3 model and RMSD values for the correspondence of the AF3 model with the experimentally determined structure in the RCSB PDB.

Crystal structures of ChlM from *Synechocystis* were obtained with either SAM or SAH bound, at resolutions of 1.6 and 1.7 Å, respectively.^112^ This monomeric, soluble protein has a central seven-stranded β-sheet, with the predicted strand order and conformation,^110^ flanked by 8 α-helices; structural differences were noted for ChlM–SAH and ChlM–SAM.^112^ In the absence of a structurally defined bound MgPIX substrate, it was docked *in silico* and the binding pocket was tested using mutagenesis, isothermal calorimetry and functional assays. These analyses showed that Tyr-28 and His-139 were necessary for catalysis, and modelling showed the C13 propionate group of MgPIX lies close to the hydroxyl of Tyr-28, the Nε nitrogen of His-139, and the *S*-methyl group of SAM.^112^ It was suggested that the C13 propionate carboxyl, positioned by Tyr-28, is deprotonated by His-139, creating the conditions for nucleophilic attack by the carboxyl on the SAM *S*-methyl group.^112^ Furthermore, the identification of two flexible arms of ChlM could explain earlier observations of random binding and enzyme isomerisation.^110,111^ The AF3 model of the SAM–MgPIX–ChlM ternary complex in Fig. 6 agrees with this previous crystallographic and *in silico* modelling study, placing the C13 propionate of MgPIX within 3.8 Å of the SAM methyl group (Fig. 6D). No obvious Mg coordinating residues were identified in our model although a water, mediated by the backbone carbonyl of Phe219 or the hydroxyl oxygen of Ser185, could provide a coordination bond, but it is not modelled here. Fig. 6C depicts the surface hydrophobicity of the methyltransferase, with an open substrate cleft flanked by hydrophobic residues (in gold), providing a possible hydrophobic interface with the underlying membrane bilayer, and a path for substrate and product tetrapyrroles to enter and leave the enzyme active site *via* the membrane bilayer. This topic is covered in more detail in Section 6 and Fig. 15.

#### 4.2.1. Linkage between the magnesium chelation and methytransferase steps

There were indications from kinetic studies that the ChlH subunit of MgCh stimulates catalysis by ChlM, and a catalytic intermediate had been found that forms before the MgDME product.^111^ Subsequently it was found that ChlH shortens the lag phase that precedes MgDME evolution, and rate constants for the accumulation of MgDME improved from 11.8 s^−1^ to 31.7 s^−1^ in the presence of 2 µM ChlH.^113^ The ChlH concentration at half the maximal rate of product formation, 1.2 ± 0.3 µM, was suggested to represent the binding constant (*K*_D_) for the binding of ChlH to ChlM.^113^ It is possible that ChlH directly accelerates the methytransferase reaction chemistry, and the ChlM structure indicated that ChlH could interact with the flexible N-terminal and G arms of ChlM,^112^ thereby mediating the stimulatory effect of ChlH on the methytransferase reaction. Further control and regulation of the coupling between chelatase and methyltransferase steps could be exerted by Gun4, which preferentially binds MgDIX over DIX (*K*_D_ 0.3 ± 0.02 µM *vs.* 2.29 ± 0.28 µM, respectively^102^). The *Synechocystis* Gun4–MgDIX structure shows an exposed ring C propionate, the methylation target for ChlM, pointing at a possible substrate delivery mechanism for the porphyrin.^107^

### 4.3. Step 3 – magnesium–protoporphyrin IX monomethyl ester [oxidative] cyclase

The formation of the isocyclic ring E is crucial for all Chls and BChls in several respects. As noted earlier, the stiffening of the macrocycle by this fifth ring enforces coplanarity of the C-13^1^ C=O group with the macrocycle,^9^ selectively delocalising the conjugated π-system along the Q_*Y*_ axis and redshifting the associated absorption band. Thus, the red cyclase substrate, MgPME, is converted to a green product, 8V-PChlide *a* (Fig. 4) *via* an oxygen-requiring reaction. Two types of MgPME cyclase have evolved, with oxygenic phototrophs such as cyanobacteria, algae and plants, as well as some purple bacteria, using molecular oxygen,^114,115^ whereas most anoxygenic phototrophic bacteria employ a mechanistically different cyclase that forms the 13^1^-oxo group using the oxygen atom from water.^116^ Sourcing oxygen from water allows the cyclase reaction to proceed in anaerobic environments, catalysed by an oxygen-sensitive radical SAM enzyme with [4Fe–4S] and cobalamin cofactors.^117^

This section will focus on the O_2_-dependent cyclase, which falls into three classes^118^ that all possess a core catalytic subunit, AcsF; this is the only cyclase component in the Betaproteobacteria, Gammaproteobacteria, Acidobacteria and Chloroflexi, whereas an auxiliary subunit is required by the Alphaproteobacteria (BciE)^119^ and the oxygenic phototrophs (Ycf54).^120–123^ AcsF was first identified in *Rubrivivax* (*Rvi*.) *gelatinosus*,^124^ followed by homologues in *C. reinhardtii*,^125^*Synechocystis*,^126–128^*Arabidopsis thaliana* and barley (*Hordeum vulgare*).^129^

Eventually, it became possible to measure the kinetics of the O_2_-dependent cyclase using the purified protein.^130^ Recombinant AcsF was produced in *E. coli* as a single, ∼44 kDa polypeptide containing 2.35 ± 0.04 iron atoms per monomer, which oligomerised to form dimers or trimers; absorption spectra indicated the presence of an µ-oxo-bridged di-iron cluster. A continuous assay was developed for the cyclase, by combining purified AcsF with NADPH, ferredoxin (Fd) and Fd:NADP^+^ reductase (FNR); measurements of reaction kinetics revealed a turnover rate of 0.9 min^−1^, a *K*_M_ for MgPME of 7.0 µM and a *K*_D_ for MgPME of 0.16 µM.^130^ Liquid chromatography–electrospray ionization–tandem mass spectrometry (LC–ESI–MS/MS) established that formation of the 8V-PChlide *a* product proceeds *via* 13^1^-hydroxy–MgPME and 13^1^-keto–MgPME, each of which successively evolved then decayed during the cyclase assay in the manner of reaction intermediates.^130^

The catalytic cycle consists of three sequential reactions, each involving the supply of two electrons by NADPH *via* FNR and the Fd carrier.^130^ The turnover rates of the chelatase (0.8 min^−1^) and cyclase (0.9 min^−1^) enzymes are similarly slow relative to the intervening methyltransferase step (3420 min^−1^), and both reflect the difficult chemistries involved. Cyclisation involves three, two-electron transfers coupled to three successive activations of molecular oxygen by active site iron atoms, in each case *via* a reactive diiron(iv)-bis–oxo intermediate.^131^ Formation of the 13^1^-hydroxy–Mg PME intermediate will consist of a multistep catalytic cycle requiring the concerted supply of electrons and molecular oxygen, followed by another cycle forming the 13^1^-keto–Mg PME, then yet another that generates the final 8V-PChlide *a* product. It was possible to couple the cyclase assay to a reconstituted PSI electron transport system, so in oxygenic phototrophs the ultimate source of electrons for the cyclase *via* reduced Fd is likely light-driven charge separation in this complex.^130^

**Fig. 7:**
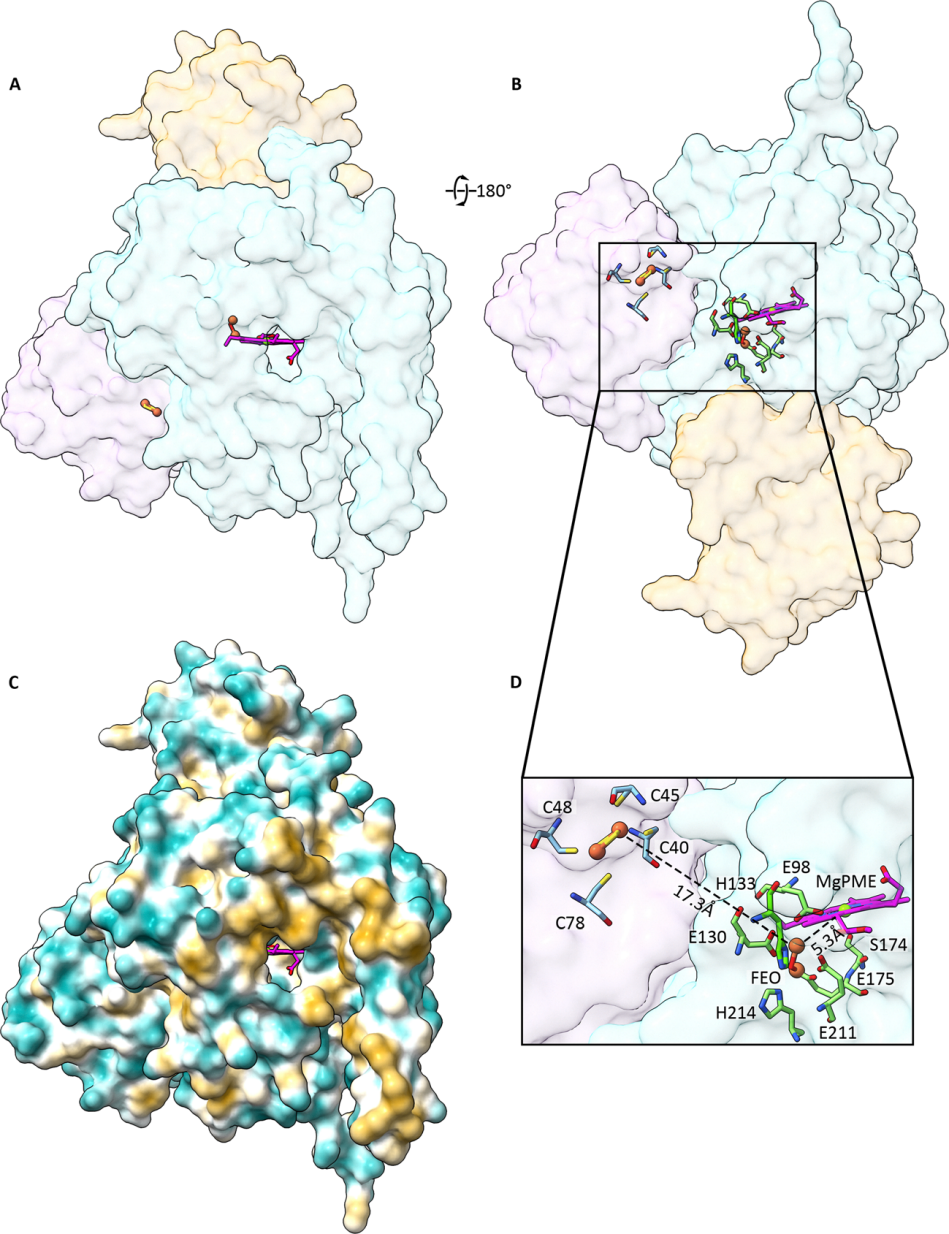
AF3 model of the Mg–protoporphyrin IX monomethyl ester cyclase complex formed from AcsF1 (light blue), Ycf54 (pastel orange) and Fd (light pink). (A) and (B) Two surface views of the overall topology of the cyclase complex with Mg–PME shown in magenta (heteroatom colours: blue – nitrogen, red – oxygen), a µ-oxo-bridged diiron cluster (heteroatom colours: red – oxygen, orange – iron) and the [2Fe–2S] cluster (heteroatom colours: yellow – sulfur, orange – iron). (C) Surface hydrophobicity (gold – hydrophobic, blue – hydrophilic) showing an open substrate binding cleft for AcsF1 flanked by hydrophobic residues. (D) Expanded view of the active site highlighting important residues involved in binding the µ-oxo-bridged diiron cluster – E98, E130, H133, E175, E211 and H214. A putative weak Mg–PME coordinating residue, S174, is also shown. The black dashed lines indicate distances from the [2Fe–2S] cluster to the diiron center and from the diiron center to the C13^1^ carbon of MgPME, which are 17.3 Å and 5.3 Å, respectively. See Table S1 in the SI for ipTM scores for the AF3 model and RMSD values for the correspondence of the AF3 model with any experimentally determined structure in the RCSB PDB.

In the absence of a structure for AcsF we used AF3 to generate a model, which has a core comprising a four-helix bundle incorporating a di-iron center held by H133, E198, E130, E211, H214 and E175 ligands (Fig. 7), while molecular oxygen forms the remaining two ligands to the di-iron cluster (not shown). Fd is also modelled, suggesting the [2Fe–2S] cluster is ∼17 Å from the di-iron center, within electron transfer distance. Of the nine Fds in *Synechocystis*,^132^ the one modelled here is the [2Fe–2S] PetF (Ssl0020). The di-iron cluster is ∼5 Å from the substrate C13^1^ atom but an intervening molecular oxygen (Fig. 7D) would be 3.6 Å from C13^1^. This arrangement closely aligns with the di-iron catalytic core of methane monooxygenase hydroxylase from *Methylococcus capsulatus*.^133^ Ser174 is ∼2 Å from the central Mg^2+^ ion, which suggests it may provide a weak coordination bond.

Oxygenic phototrophs require an auxiliary cyclase subunit, which is Ycf54 in *Synechocystis*^122,134^ and LCAA in tobacco.^135^ This small, soluble protein of 12.5 kDa is essential for the assembly and function of the cyclase complex, and inactivating *slr1780* encoding Ycf54 in *Synechocystis* greatly lowers Chl and PChlide levels in the cell, while accumulating the cyclase substrate MgPME.^122^ Although full segregation of a *ycf54* deletion mutation could not be achieved, it was nevertheless clear that Ycf54 is required to form an active cyclase, although there were no bound cofactors or a likely catalytic site in structures of Ycf54 homologues deposited in the PDB (PDB 3HZE; *Thermosynechococcus elongatus*, and PDB 3JSR; *Nostoc* sp. PCC 7120). A wider influence on the early stages of Chl biosynthesis was suggested because lowered amounts of Ycf54 in the *ycf54* mutant were accompanied by decreases in the levels of the AcsF1 subunit, as well as the MgPIX methyltransferase and PChlide oxidoreductase.^122^

The Ycf54–AcsF1 interaction has been investigated using structural and functional methods. Sequence alignments of Ycf54 homologues from a wide range of oxygenic phototrophs revealed a core domain of 90 residues, seven of which, A9, F13, E22, E26, D39, F40 and R82 (*Synechocystis* numbering) are particularly highly conserved. Mutating these residues showed that A9G decreases the amounts of Ycf54 and AcsF1, while R82A lowered Chl levels, abolished interaction with the AcsF1 catalytic subunit, and impaired photosynthetic growth.^121^ D39A and F40A mutants also stopped the AcsF1–Ycf54 interaction. To investigate the Ycf54–AcsF1 interaction further, crystal structures were solved for Ycf54 from *Synechocystis* (5M2P, 1.3 Å), and for the R82A (5M2U, 2.2 Å) and A9G (5M2R, 1.5 Å) mutants. Ycf54 comprises a single domain consisting of a central four-stranded antiparallel β-sheet (β1–β4) flanked by α1, α2 and α5 helices on one side and by helices α3 and α4 on the other, and these features were maintained in the R82A and A9G variants. However, R82A had lost stabilising hydrogen bonds with neighbouring W78, F20 and E17 residues, and the surface electrostatics on this part of the wild-type and R82A structures changed from being positive overall to predominantly negative. It was speculated that the flexibility and positive charge of R82 are required for docking of Ycf54 onto AcsF1.^121^ AF3 modelling predicts that R82 interacts directly with AcsF1 *via* a salt bridge with D216. A9 lies at the end of the first β-strand and interacts internally with Ycf54 residues L14 and F15; the A9G mutant likely affects L14 and L15, which form part of the interface with AcsF1. The conserved residue D39 of Ycf54 is predicted to associate internally with Y31, which is found on a helix that directly interacts with AcsF1, and conserved residue F40 may form a π–cation interaction with R54 on the same helix. Thus, AF3 modelling shows how mutation of several conserved residues in Ycf54 might disrupt its interface with AcsF1.

### 4.4. Step 4 – light-dependent protochlorophyllide oxidoreductase

In this biosynthetic step reduction of the ring D double bond at C17–C18 forms a chlorin, which increases the relative amplitude of Q_*Y*_ absorption as well as redshifting the maximum from 629 nm to 666 nm, close to the absorption maximum for Chl *a* (Fig. 4). Nature has found two contrasting ways of reducing this double bond: anoxygenic phototrophic bacteria use a PChlide oxidoreductase (POR) enzyme that is mechanistically and structurally related to nitrogenase, the multi-subunit, ATP-requiring metalloenzyme that reduces nitrogen to ammonia. As with nearly all enzymes, this type of POR does not require light to initiate its catalytic cycle, but it is called light-independent POR or DPOR (EC 1.3.7.7) to distinguish its operation in the dark from the wholly different light-dependent enzyme, (LPOR, E.C. 1.3.1.33). Crystal structures of DPOR have been reported,^136–138^ and the following reviews summarise the structure, function and biological role of this enzyme.^6,8,58,139,140^

LPOR is used by cyanobacteria, algae and plants, all of which generate oxygen as a by-product of water splitting by PSII,^141^ thus creating problems for a nitrogenase-based DPOR enzyme.^142^ There are several isoforms of LPOR in plants, which have been studied *in vitro*,^58,143,144^ but the structural modelling in this section the focusses on the LPOR of cyanobacteria, specifically *Synechocystis*. The literature on POR exceeds that for any other Chl biosynthesis enzyme, partly because the POR photocycle is intertwined with the wider topic of photomorphogenesis in plants.^58,145^ Thus, the development and assembly of photosynthetic membranes is held back in the dark so POR, together with its PChlide and NADPH substrates, accumulates in etiolated plant tissues within proplastids (etioplasts) forming prolamellar bodies (PLBs).^146–149^ PLBs are remarkable, tubular paracrystalline structures, which rapidly dissemble when light-dependent turnover of POR converts PChlide to Chlide,^150–152^ with the eventual formation of the lamellar thylakoid membranes that house photosynthetic complexes for light harvesting and charge separation.^153^

The other large body of research on POR relates to its value as a model system, because LPOR is one of a select group of enzymes that use light to trigger catalysis.^154–158^ Thus, mechanistic research on POR has transcended the field of Chl biosynthesis, and kinetic and structural studies of LPOR have revealed new and generally important information on the rates and mechanisms of enzymatic hydride, proton and electron transfers.^156,159,160^ As with the other Chl pathway enzymes, the availability of large quantities of pure, active recombinant protein, from a cyanobacterial source, was decisive.^161^ Early cryo-trapping experiments with mesophilic and thermophilic LPORs from *Synechocystis* and *T. elongatus*, respectively, showed that the initial light-driven reaction of the ternary NADPH–PChlide POR complex could occur even at 120 K, followed by a series of stepwise ‘dark’ reactions that could either proceed or be halted by careful temperature control, implying the involvement of domain movements and/or reorganization of the protein on release of the NADP^+^ and Chlide products, then also for binding of another round of NADPH and PChlide substrates.^154,155,162,163^ Early ultrafast absorption experiments with 50 fs laser pulses showed that product states appear on a picosecond timescale,^164^ but more detailed ultrafast studies were required to dissect the hydride, proton and electron transfers that collectively reduce the C17–C18 double bond.^156,159,160^

Structures were clearly essential to formulate a coherent mechanistic view of the LPOR catalytic cycle, and an early model of LPOR from *Synechocystis*, based on its homology to the family of short-chain dehydrogenase and reductase (SDR) enzymes, was a useful guide.^165^ Then, crystal structures of cyanobacterial NADPH–LPOR complexes from *Synechocystis* and *T. elongatus* were determined;^166,167^ although PChlide was absent, a series of mutagenesis and docking studies positioned PChlide within a binding pocket, and a LPOR–NADPH–PChlide ternary complex was proposed.^167^ Currently, the only structural studies that include both the NADPH and PChlide substrates are cryo-EM analyses of tubular LPOR assemblies from *A. thaliana*; these tubular filaments can be assembled *in vitro* from purified lipid, protein and substrate components, and are used as a convenient proxy for the more intricate PLBs found *in vivo*, in which interconnected tubules form a 3D cubic lattice.^152,168^ Thus, such work brings together the two notable features of LPOR, namely its crucial role in photomorphogenesis and thylakoid membrane formation, and its value as a structurally defined, kinetically accessible model enzyme system.

**Fig. 8:**
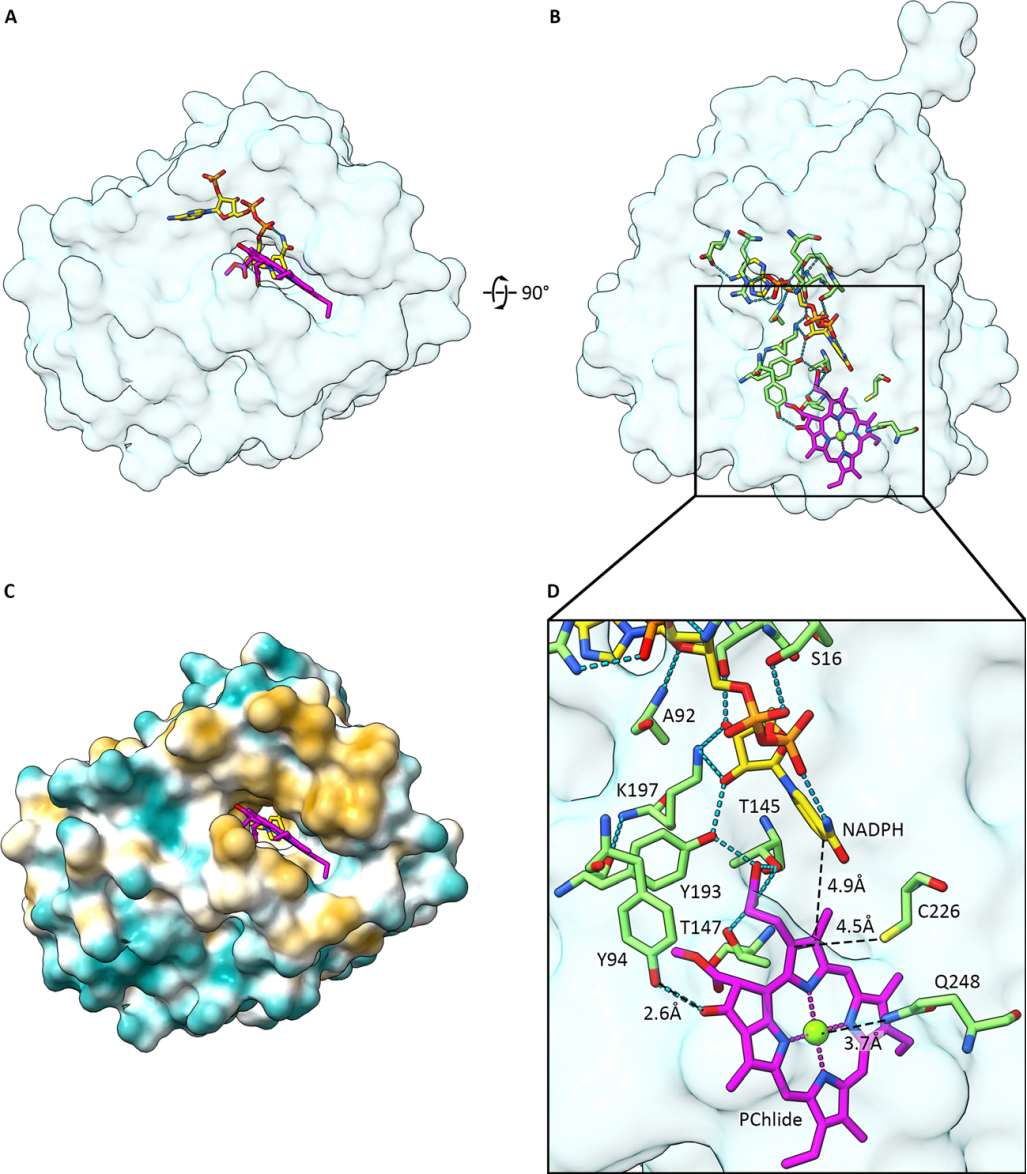
AF3 model of the light-dependent PChlide oxidoreductase. (A) and (B) Two surface views of the overall topology of the protein with 8V-PChlide and NADPH shown in magenta and yellow, respectively (heteroatom colours: blue – nitrogen, red – oxygen, orange – phosphate). (C) Surface hydrophobicity (gold – hydrophobic, blue – hydrophilic) showing an open substrate binding cleft for LPOR flanked by hydrophobic residues. (D) Expanded view of the active site highlighting important residues involved in binding NADPH and 8V-PChlide, and some residues implicated in catalysis. The backbone amino nitrogen of A92 interacts with the adenosine ribose ring oxygen of NADPH; the alcohol group of S16 interacts with one of the bridging phosphate groups closer to the adenosine moiety; K197 and Y193, previously implicated in reducing the C17=C18 double bond, interact with the alcohol groups of the nicotinamide ribose. Y193, T145 and T147 appear to form interactions with the C17 propionate carboxyl of 8V-PChlide; Y94 interacts with the 13^1^-keto group of 8V-PChlide; Q248 may provide a weak co-ordination bond with the central Mg *via* the amide nitrogen. The distance from the C4 group of the hydride donor NADPH is 4.9 Å from the C18 carbon and C226 is 4.5 Å from the C17 carbon. See Table S1 in the SI for ipTM scores for the AF3 model and RMSD values for the correspondence of the AF3 model with experimentally determined structures in the RCSB PDB.

The structural model of the LPOR–NADPH–PChlide ternary complex from *Synechocystis* (SynLPOR) in Fig. 8, calculated using AF3, closely resembles the cryo-EM structure of the plant complex (AraLPOR) (7JK9;^152^), rather than the PChlide-free cyanobacterial structures (6L1G (91) and 6R48 (92)). There is close agreement for all structurally determined sites for binding NADPH,^152,166,167^ but not for PChlide. In both the AF3 SynLPOR model and the AraLPOR structure PChlide sits in a cavity, free from the obstructing loop present in 6R48 and 6L1G; instead, this membrane-facing region of POR is rearranged, and the loop moves to one side to admit PChlide, with the α-10 helix on the distal side (not shown in Fig. 8). The opening to the PChlide binding cleft of AraLPOR lies on the membrane,^152^ effectively enclosing the PChlide, and a similar arrangement could apply to PChlide bound to SynLPOR. The AraLPOR lipid-binding residues are situated on the loop that forms the base of the PChlide binding site, opposite the α-10 helix, and the monogalactosyldiacylglycerol (MGDG) lipid head group largely binds *via* a combination of polar and non-polar residues. This suggests that the hydrophobic α-10 helix is important for interacting with the membrane bilayer, and this topic will be returned to in Section 6 because it illustrates a general point about the way Chl biosynthesis enzymes might interact with the underlying membrane and with one another.

The differing positions of the loop and α-10 helix in the SynLPOR (AF3)/AraLPOR (7JK9) ternary complexes compared with the SynLPOR–NADPH (6R48/6L1G) structures have consequences for the PChlide binding site and reveal a new set of active site residues. Interestingly, the SynLPOR–NADPH structures (6R48/6L1G) may represent a state in the catalytic cycle where the α-10 helix points into the photosynthetic membrane to “fish” for substrates. In relation to the AF3 model, we note that Q248, found on the α-10 helix, is 3.7 Å from the central Mg, potentially acting as a transient ligand to the PChlide substrate. Two moieties are close to the C17–C18 double bond on ring D (Fig. 8D); the nicotinamide ring of NADPH is 5.4 Å from C17 and 4.9 Å from C18, and the corresponding distances for C226 are 4.5 Å and 3.9 Å, respectively. Thus, based on distance alone, and bearing in mind their modelled inaccuracies, the hydride from NADPH and the proton from C226 could attack either carbon. The C226S mutant has only 7% of wild-type activity,^169^ and proton transfer for the C226A variant is > 200-fold slower than for wild-type LPOR.^158^ Y193 and K197 were originally assigned as a catalytic motif, based on their conserved roles in the SDR family,^154,165,167^ and are essential for activity,^170–172^ but here Y193 and K197 interact with the NADPH ribose oxygens and Y193 with the C17 propionate group of PChlide. The model highlights other PChlide-interacting residues which may be important for ensuring correct orientation of the macrocycle and access to the C17–18 bond by the hydride. Y94 is predicted to interact with the C13^1^ keto group of PChlide, while T147 and T145 interact with the propionate group oxygens.

Once PChlide is established within the active site, transiently liganded by Q248, oriented by interactions with LPOR side chains such as Y94, Y193, T147 and T145, and with the C17=C18 bond held adjacent to C226 and the NADPH nicotinamide ring, the ternary complex is primed for catalysis. The POR catalytic cycle is initiated when PChlide absorbs a photon, and a charge transfer state^157^ polarises the C17=C18 double bond, predisposing it to hydride transfer from the nearby NADPH. Several possibilities exist for hydride transfer, discussed in ref. 173, and it appears that this multi-step process proceeds in about 500 ns *via* an initial electron transfer followed by a hydrogen (proton plus electron) transfer.^159,160^ The next step involves microsecond proton transfer from C226 to conclude reduction of the C17=C18 bond. More detailed discussions are beyond the scope of this summary and can be found in ref. 158, 173 and 174. The product release steps have been investigated,^162,163^ and in angiosperms they are likely associated with the rapid dissolution of PLBs,^151^ proposed to be initiated when Chlide forms.^152^ The geometry at C17 changes upon reduction, from trigonal to tetrahedral, which displaces the propionate out of plane, and in angiosperms this movement is proposed to push the α-10 helix, leading to dissociation of LPOR from the membrane.^152^

### 4.5. Step 5 – 8-vinyl reductase

The majority of phototrophs convert 8V-Chlide *a* to 8E-Chlide *a* (Chlide *a* in Fig. 4, but also called MV-Chlide *a*), which enhances the absorption of red wavelengths by the Q_*Y*_ band (Fig. 4), although some marine cyanobacteria retain the 8V group.^12,21^ There are several types of 8V reductase (8VR, also called divinyl reductase, DVR); a gene encoding an 8VR, AT5G18660, was discovered in *Arabidopsis*,^175,176^ and 8VR activity was confirmed following recombinant production in *E. coli*.^175^ Homologues of the protein encoded by AT5G18660, now called BciA, have been found in rice,^177^*Rba. sphaeroides*^178^ and green sulfur bacteria,^179^ for example. Whereas BciA is an NADPH-requiring enzyme,^179^ there is another type of 8VR, BciB, that obtains electrons from reduced Fd. The recombinant BciB from the green sulfur bacterium *Chloroherpeton thalassium* has a flavin adenine dinucleotide (FAD) cofactor and two [4Fe–4S] clusters.^180^ Among the cyanobacteria, *A. marina* is unusual in having both BciA and BciB,^181^ and in *Synechocystis*, the main subject of this review, formation of the 8-ethyl group is catalysed only by BciB.^182,183^

**Fig. 9:**
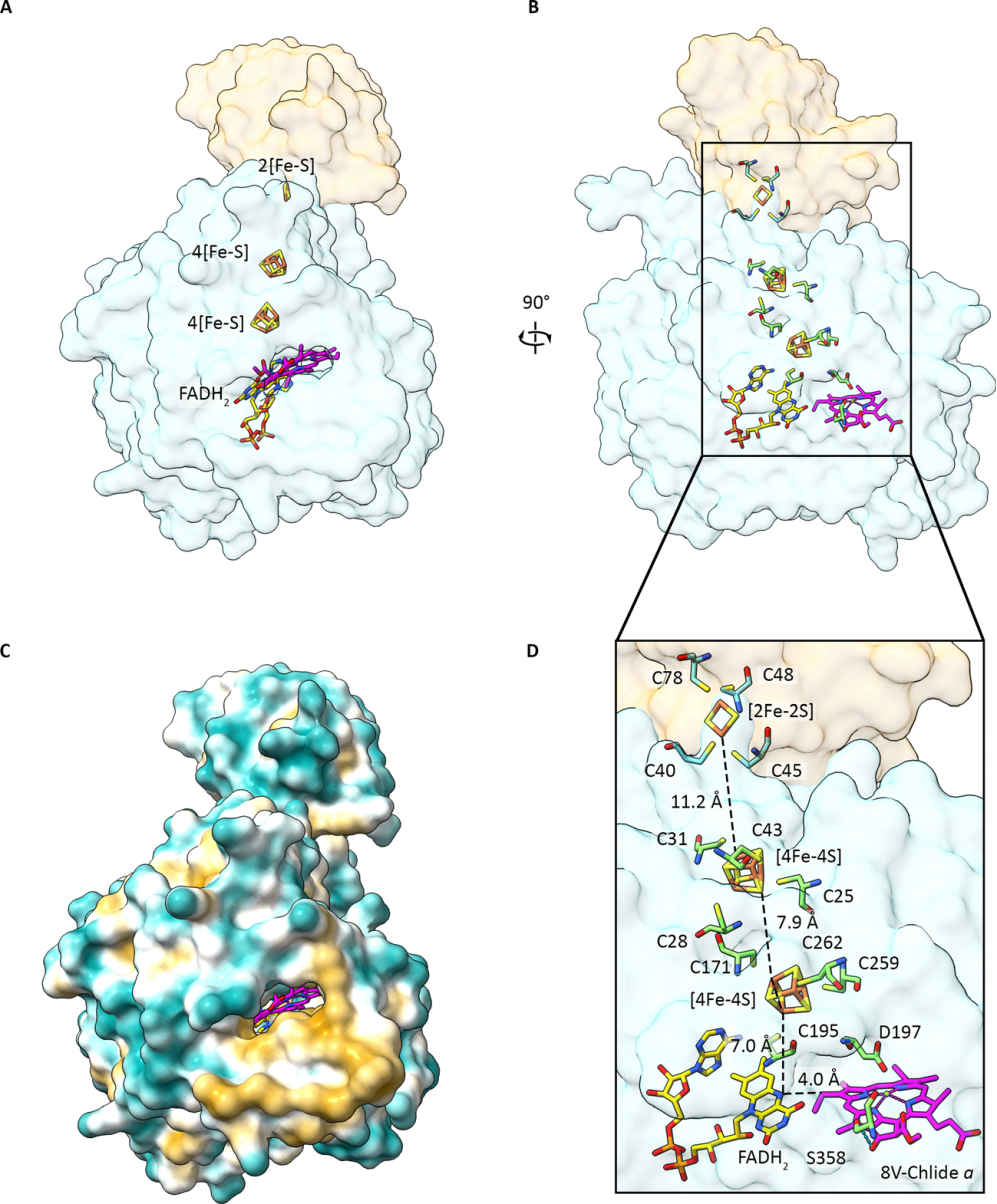
AF3 model of the 8V reductase in complex with Ferredoxin, Fd, which provides the electrons for the reduction reaction, along with its cofactors, two [4Fe–4S] clusters and FADH_2_. (A) and (B) Two surface views of the overall topology of the protein with 8V-Chlide in magenta (heteroatoms coloured: blue – nitrogen, red – oxygen). Fe–S clusters are coloured by heteroatom: dark orange – iron, yellow – sulfur. FADH_2_ is shown in yellow (heteroatoms coloured: blue – nitrogen, red – oxygen, orange – phosphate). These panels also highlight the relative location of the Fd [2Fe–2S] cluster, the 8VR [4Fe–4S] clusters, the FADH_2_ cofactor and the 8V-Chlide *a* substrate. (C) Surface hydrophobicity (gold – hydrophobic, blue – hydrophilic) showing an open substrate binding cleft for 8V reductase flanked by hydrophobic residues. (D) Expanded view of the active site highlighting the cysteine residues involved in coordinating the Fe–S clusters. In Fd, C40, C45, C48 and C78 coordinate a [2Fe–2S] cluster. In 8VR, the [4Fe–4S] cluster proximal to the Fd binding site is coordinated by C25, C28, C31 and C43, and the [4Fe–4S] cluster that reduces the vinyl group is coordinated by C171, C195, C259 and C262. Residues that interact with FADH_2_ have been omitted for clarity. The black dashed lines indicate various distances: the Fd [2Fe–2S] cluster to the first 8VR [4Fe–4S] cluster (11.2 Å), the first 8VR [4Fe–4S] cluster to the second (7.9 Å), the second [4Fe–4S] cluster to the FADH_2_ nitrogen (7.0 Å), and from the FADH_2_ nitrogen to the 8V group of 8V-Chlide *a* (4.0 Å). Two residues interacting with the macrocycle substrate were also identified; S358 provides a hydrogen bond to the C13^1^ keto group and D197 may provide a coordination bond to the central Mg^2+^ ion. See Table S1 in the SI for ipTM scores for the AF3 models and RMSD values for the correspondence of models with any experimentally determined structures in the RCSB PDB.

The AF3 structure of *Synechocystis* BciB (Slr1923) is shown in Fig. 9. AF3 places an FAD and two [4Fe–4S] clusters within this enzyme, consistent with earlier experimental work,^180^ and the model also shows a Fd bound *via* a positively charged patch on BciB in an area formed by residues 9–40. Many of these interactions are between backbone atoms, but the H37 and K22 side chains are predicted to interact with Fd. We used the [2Fe–2S] PetF (Ssl0020), the same Fd modelled in the cyclase complex (Section 4.3), which docks with its [2Fe–2S] center 11.2 Å from the proximal [4Fe–4S] cluster of BciB. For comparison, the distance between the [2Fe–2S] center of Fd to the [4Fe–4S] F_B_ acceptor of Photosystem I is 11.7 Å.^184^ Subsequent electron transfers in BciB, between the [2Fe–2S] centers, and then to the redox active FADH_2_ isoalloxazine nitrogen, are over distances of 7.9 Å and 7.0 Å, respectively. The final step transfers a hydride from FADH_2_ which is 4.0 Å from the C8 vinyl group (Fig. 9D). The AF3 model also reveals two residues predicted to interact with the substrate macrocycle; D197 provides a putative weak coordination bond to the central Mg of 3,8-divinyl Chlide *a* and there is a hydrogen bond from S358 to the C13^1^ keto group. Features of the AF3 model of BciB are found in the FrhB subunit of the F_420_-reducing [NiFe]-hydrogenase from *Methanothermobacter marburgensis*,^185^ which has sequence homology with BciB.^180^ FrhB also has a [4Fe–4S] cluster, an FAD, and a binding site for a tetrapyrrole substrate, coenzyme F_420_.

### 4.6. Step 6 – chlorophyll synthase

ChlG is the only a transmembrane enzyme in the Chl biosynthesis pathway, which reflects the large increase in hydrophobicity when the C17-propionate of the Chlide macrocycle is esterified by the C20 diterpenoid geranylgeraniol. Early studies had identified and assigned *bchG* genes encoding the BChl synthase in purple bacteria,^53,55^ and ChlG homologues followed, with overexpression in *E. coli* yielding active Chl synthase.^186^ The hydrophobic nature of this enzyme and the inability to purify sufficient quantities for structural and kinetic studies delayed progress, but it was found that a ∼83 kDa ChlG_2_–HliD_2_ complex accumulates in a mutant strain of *Synechocystis*.^187^ This complex could be purified and structure determination by cryo-EM yielded both apo (3.0 Å resolution) and GGPP-bound (3.2 Å) forms of the enzyme.^188^ The overall structure comprises a central HliD dimer flanked on each side by a ChlG monomer consisting of nine TMHs that form a large substrate-binding cavity gated by cytoplasmic entry loops. These structural features have been found in other prenyltransferases,^189,190^ and the similarities likely extend to their catalytic mechanisms. It was already known that ChlG associates with HliD, a high light-inducible protein (Hlip), and with the membrane protein insertion machinery,^191^ but this work provides the first structure of the photoprotective ChlG_2_–HliD_2_ complex. This advance also aids understanding of the HliD-mediated photoprotection of Chls delivered to the machinery for inserting nascent photosystem polypeptides into membranes.

**Fig. 10:**
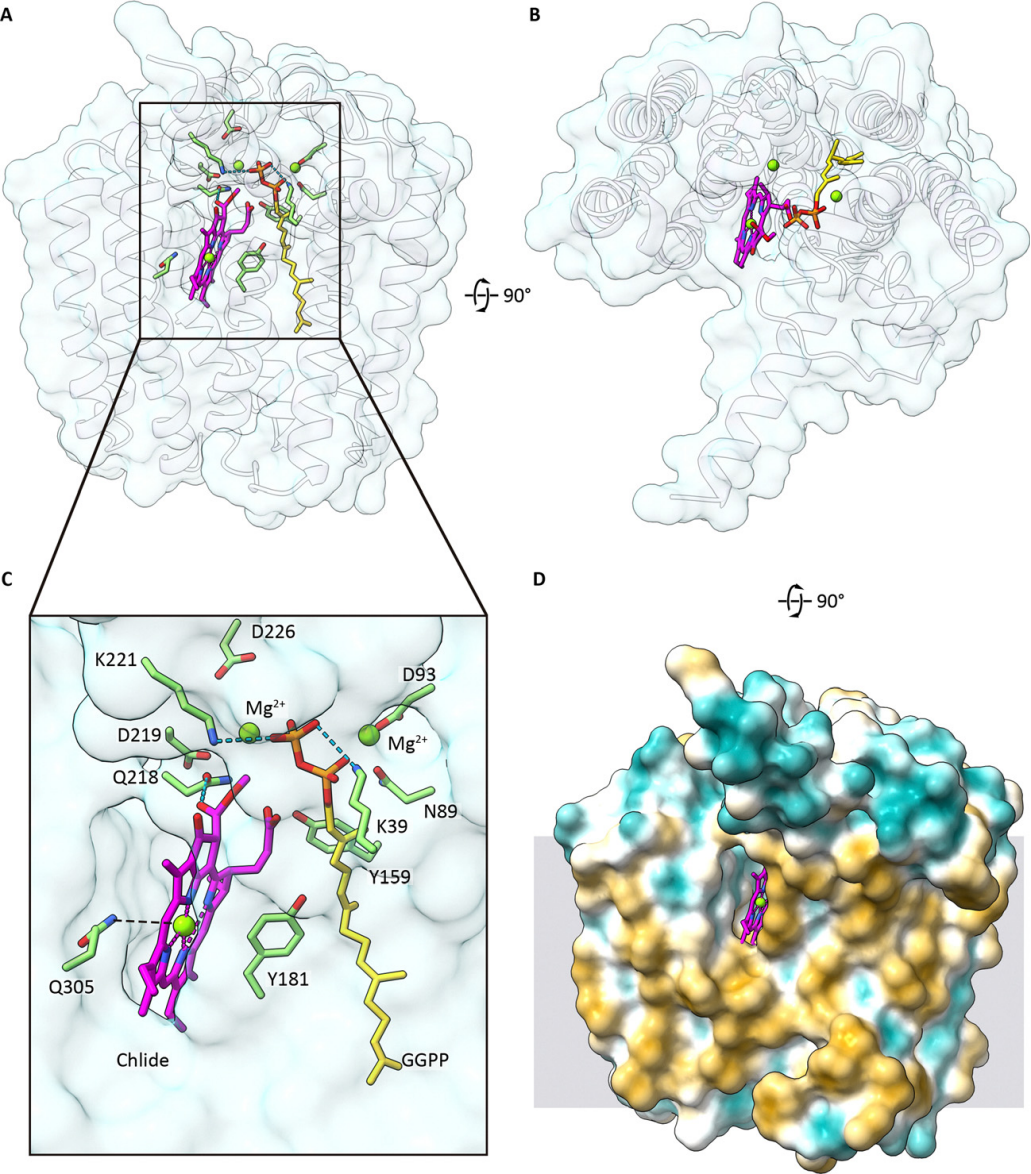
AF3 model of chlorophyll synthase in a ternary complex with its substrates Chlide *a* and GGPP. (A) and (B) Two surface views of the overall topology of the protein with Chlide *a* and GGPP in magenta and yellow, respectively (heteroatoms coloured: blue – nitrogen, red – oxygen, orange – phosphate). (C) Expanded view of the active site, showing several residues involved in substrate binding and activation. D93 and N89 coordinate a catalytic Mg^2+^, which interacts with the pyrophosphate group; D219 and D226 are hypothesised to play the same role with another Mg^2+^. K39 and K221 may also play an activating role, but at minimum they provide structural stability to the pyrophosphate group. Y159 and Y181 likely stabilise the ∂^+^ terminal GG carbon. Q218 interacts with the C13^2^ ester group. Q305 provides a weak ligand to the central magnesium ion of Chlide *a*. (D) Surface hydrophobicity (gold – hydrophobic, blue – hydrophilic) showing the binding cleft for the Chlide *a* substrate flanked by hydrophobic residues, and open to the membrane interior. The distribution of hydrophobic residues is consistent with indicating the transmembrane nature of ChlG – a cartoon representation of the membrane is shown in grey. See Table S1 in the SI for ipTM scores for the AF3 model and RMSD values for the correspondence of models with any experimentally determined structures in the RCSB PDB.

The cryo-EM structure of the ChlG_2_–HliD_2_ complex lacks Chlide bound within the active site, so AF3 was used to model the ChlG–GGPP–Chlide ternary complex^188^ (Fig. 10). There is close agreement between the cryo-EM and AF3 models (RMSD = 1.69 Å), with the tetraprenyl chain of GGPP similarly positioned in a hydrophobic cleft in both cases, but in the AF3 model Chlide *a* (magenta), two catalytically important Mg^2+^ ions (green) and GGPP (yellow) are all present. Fig. 10A and B displays the overall semi-transparent shape of the ChlG monomer enclosing the transmembrane helices, and Fig. 10C shows the active site in more detail. Q218 interacts with the C13^2^ ester group, and Q305 coordinates the central Mg of Chlide, similar to the function of Q248 in POR; such coordination bonds help to position the macrocycle within an active site but are presumably weak enough to allow product release.

The esterifiable C17 carboxyl group of Chlide *a* is positioned next to the pyrophosphate of GGPP (orange), which is stabilised by K39 and K221. Nearby, a catalytic Mg^2+^ coordinated by D93 and the pyrophosphate group oxygens acts as a Lewis acid, drawing electron density away from the terminal GGPP carbon atom; D219 and D226 appear to play the same role with another Mg^2+^, which is also coordinated by pyrophosphate group oxygens on the other face of the molecule, adding to the electron withdrawing effect of the other Mg^2+^ Collectively, these two Mg^2+^ ions make the pyrophosphate a more potent leaving group.^192^ We suggest that the terminal GG carbon is primed for a concerted SN2-type attack by the strongly nucleophilic C17^3^ carboxyl group oxygen, forming an ester bond between the GG moiety and the macrocycle. The SN2 mechanism proceeds efficiently if the directions of attack on one side of the ∂^+^ terminal GG carbon and bond breakage on its distal side are linearly aligned, and the structural model^188^ (Fig. 10) indicates a near-linear angle of 165°. The reaction can therefore proceed in a concerted manner, with no need for a two-stage SN1 mechanism involving a carbocation, as seen for other prenyltransferases.^193^ Y159 and Y181 likely stabilise the ∂^+^ terminal GG carbon and transition state intermediate, respectively. Fig. 10D shows the substrate binding cleft of the enzyme with the hydrophobic surface facing out towards the membrane bilayer as expected. The membrane-intrinsic ChlG is therefore different from the other enzymes in the Chl pathway, which all have a hydrophobic face sitting on, rather than in, the membrane. This point will be discussed in section 6.

In terms of the substrate specificity of ChlG for Chlide rather than BChlide, it was shown that the I44F mutation enabled ChlG, heterologously produced in *Rba. sphaeroides*, to participate in BChl biosynthesis.^194^ We note that the AF3 model shows the B-ring of the Chlide macrocycle adjacent to I44, suggesting a link between the identity of the sidechain at position 44 and its compatibility with a chlorin with a C7=C8 double bond *versus* a bacteriochlorin with a C7–C8 single bond.^188^ Further mutagenesis, modelling and structural work should enhance our understanding of the selectivity of synthases for making either Chls or the redshifted BChls, accelerating progress towards the synthetic biology goal of engineering organisms to produce both Chls and BChls to expand the spectral range of photosynthesis;^195^ see also Section 6.1.

### 4.7. Step 7 – geranylgeranyl diphosphate reductase

Early work on spinach chloroplasts had shown that ‘free’ GGPP could be converted to PPP in the chloroplast envelope, and a second pathway and location for reducing GG was found in the thylakoid membrane. This latter reaction initially esterified Chlide *a* to form Chl_GG_, catalysed by Chl synthase, followed by reduction of the macrocycle-attached GG moiety, forming phytyl-Chl *a*.^196^ Both types of GG reduction required NADPH and proceeded in a stepwise manner *via* the DHGG and THGG intermediates. However, this work found a four-fold preference for PPP as the substrate for Chl synthase, in contrast to the synthase in etioplasts which favoured GGPP as a substrate, so the final step in Chl biosynthesis could be regarded as the attachment of PPP to Chlide. In support of this reaction sequence, PPP was found to be a better substrate for recombinant BChl and Chl synthases than GGPP.^186^ A study of repair of damaged PSII in *Synechocystis* provided evidence for de-esterification and re-esterification of Chl, with recycling of phytol and Chlide. Under these conditions at least, the substrates for Chl synthase are likely to be PPP and Chlide.^197^

Early genetic studies of *Rba. capsulatus* identified mutations in the BChl biosynthesis gene *bchP* which halted the pathway at the level of BChl_GG_,^53^ and a *bchP* mutant of *Rba sphaeroides* could be partially complemented with a gene from *Synechocystis*, assigned as *chlP*, providing evidence for strong similarities between the BchP and ChlP GGPP reductases.^198^ Recombinant *Arabidopsis* ChlP produced in *E. coli* was able to reduce GGPP to PPP as well as Chl_GG_ to phytyl-Chl, suggesting that this enzyme can be recruited to serve both the prenylquinone and Chl pathways.^199^

**Fig. 11:**
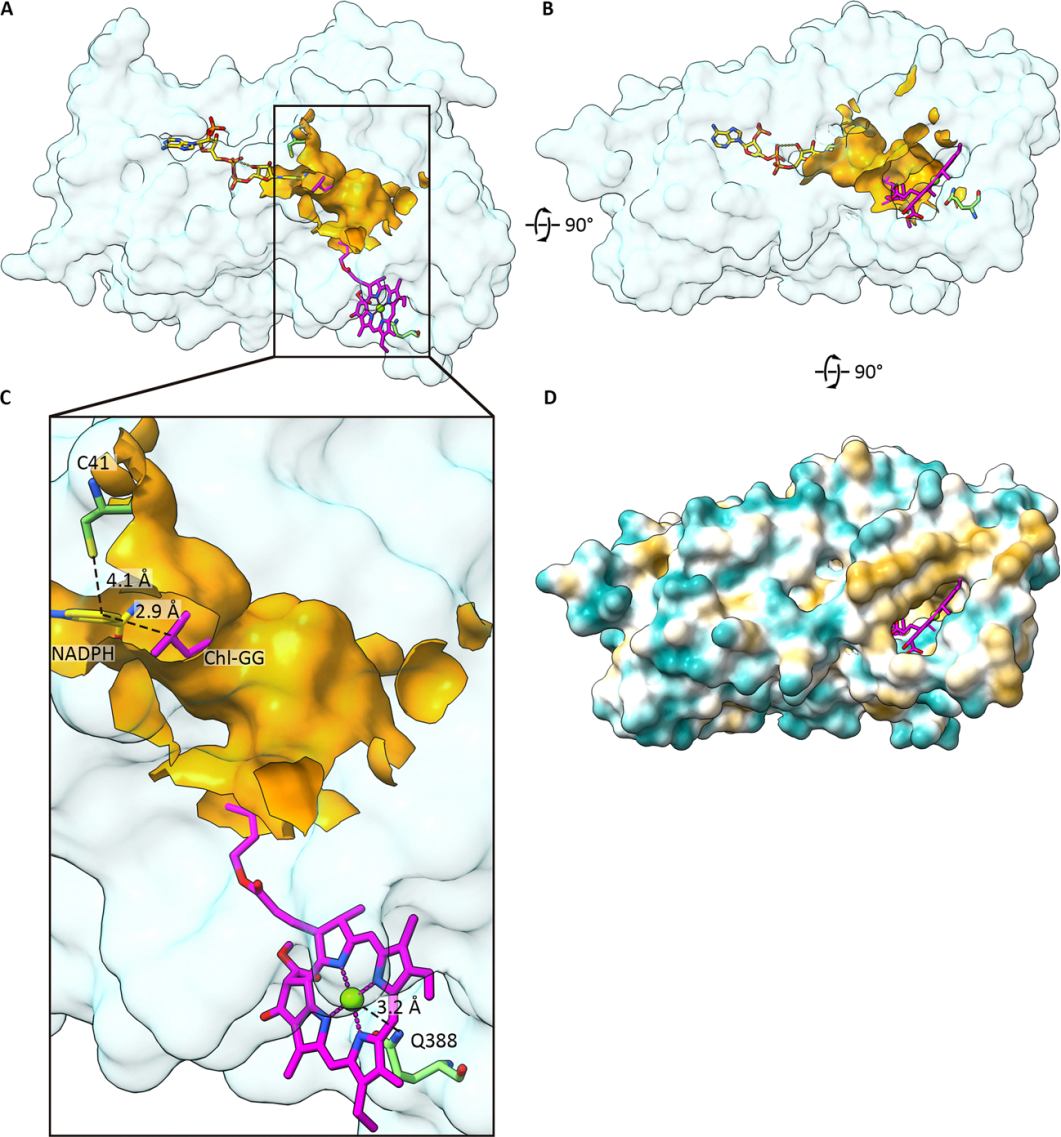
AF3 model of geranylgeranyl pyrophosphate reductase, ChlP. (A) and (B) Two surface views of the overall topology of the protein with Chl_GG_ and NADPH shown in magenta and yellow, respectively (heteroatoms coloured: blue – nitrogen, red – oxygen, orange – phosphate). (C) Expanded view of the active site, showing several residues involved in substrate binding and activation, including a potential catalytic residue, C41, and a Chl_GG_-coordinating residue, Q388. Besides these two residues, the relatively well conserved NADPH binding residues and the residues which form the GGPP binding pore, no other obvious catalytic residues were identified. The C4 group of NADPH (where the hydride is located) is 2.9 Å from the GG tail and is 4.1 Å from C41. Panels A–C also highlight a pore (orange) that runs from the substrate entry site to the C4 atom of the nicotinamide group of NADPH. It is assumed the enzyme possesses some processivity, and the GG tail is likely to move through the pore as the reductions take place. (D) Surface hydrophobicity (gold – hydrophobic, blue – hydrophilic) showing the binding cleft for the Chl_GG_ substrate flanked by hydrophobic residues. See Table S1 in the SI for ipTM scores for the AF3 model.

We modelled the structure of *Synechocystis* ChlP using AF3. Fig. 11A and B show the macrocycle of Chl_GG_ (magenta) within a cavity near a substrate entry site. Another feature seen for other Chl biosynthesis enzymes is a weak ligand to the central Mg, here formed by Q388 (Fig. 11C). The GG moiety extends away from the macrocycle into the interior of ChlP, along a pore (orange) that runs from the substrate entry site to the C4 atom of the nicotinamide group of NADPH, adjacent to a C41, which might play a catalytic role. However, there are three successive reductions along the GG tail, so the oxidised NADP^+^ formed in converting GG to DHGG must be replaced with NADPH, and once more when THGG is finally reduced to phytyl. It is assumed that GGPP reductase possesses some processivity, allowing movement of the tail through the pore to enable these three reductions of the GG tail, namely the C6–7, C10–11 and C14–15 carbon double bonds, to take place. Our modelling does not provide any insights into any mechanistically relevant conformational movements. The predicted presence of a weak coordinating ligand, and a pore large enough to admit the GGPP tail and its attached macrocycle, are consistent with Chl_GG_ as the substrate for GGPP reductase but do not exclude the same involvement for ‘free’ GGPP. So, although GGPP reductase is regarded as the final enzyme in the Chl pathway for the purpose of this review, another plausible sequence is the reduction of GGPP to PPP by GGPP reductase, and the final biosynthetic step would be esterification of Chlide by PPP, catalysed by Chl synthase.

Finally, Fig. 11D shows that the Chl_GG_ substrate gains access to the active site through a pore flanked by hydrophobic ridges, which are proposed to form a membrane interface. Similar substrate pores/cavities, flanked by hydrophobic membrane interfaces, were predicted for the methytransferase, cyclase, LPOR, 8VR, Chl *c* synthase and CAO enzymes (Fig. 6C, 7C, 8C, 9C, 13C and 14C), and this feature will be covered in more detail in Section 6.

## 5. Branches and extensions of the Chl *a* pathway – the biosynthesis of Chls *b*, *c*, *d* and *f*

Although Chl *a* is the dominant such pigment on Earth, the extensions of the main pathway to form Chls *b*, *c*, *d* and *f* are important because they allow plants, algae and cyanobacteria to colonise new ecological and spectral niches, by harvesting and using solar energy more effectively. Only one extra enzyme is apparently needed to create each Chl variant, and the structures of the respective synthases have been calculated using AF3.

### 5.1. Biosynthesis of Chls *c*_1_ and *c*_2_

Fig. 3 shows that while Chls *a*, *b*, *d* and *f* have Chlide *a* as a common intermediate, Chls *c*_1_ and *c*_2_ branch from the main pathway at the level of 8V-PChlide *a*. Brown algae, diatoms, and dioflagellates use these accessory Chls to absorb more of the blue light that filters through water columns, so they make a major contribution to marine productivity.^20,200^ A *CHLC* gene encoding chlorophyll *c* synthase was discovered in the marine diatom *Phaeodactylum tricornutum*, and recombinant CHLC protein converted 8V-PChlide and PChlide (MV–PChlide) to Chl *c*_2_ and Chl *c*_1_, respectively,^201^ in a reaction requiring Fe^2+^ and 2-oxoglutarate (2OG). The synthase gene was also found in a dinoflagellate, *Breviolum minutum*, and its function was confirmed by heterologous expression in *Nicotiana benthamiana*.^202^ Here, the synthase has a predicted but dispensable Chl *a*/*b* binding domain, and an essential catalytic domain belonging to the superfamily of 2OG-Fe(ii) dioxygenases.^203^ These catalytically flexible enzymes use 2OG and O_2_ to oxidise a wide variety of substrates, most commonly hydroxylations but also desaturations,^204^ as seen for CHLC, which forms an acrylate by introducing a C17^1^–C17^2^ double bond into the C17 propionate.

**Fig. 12:**
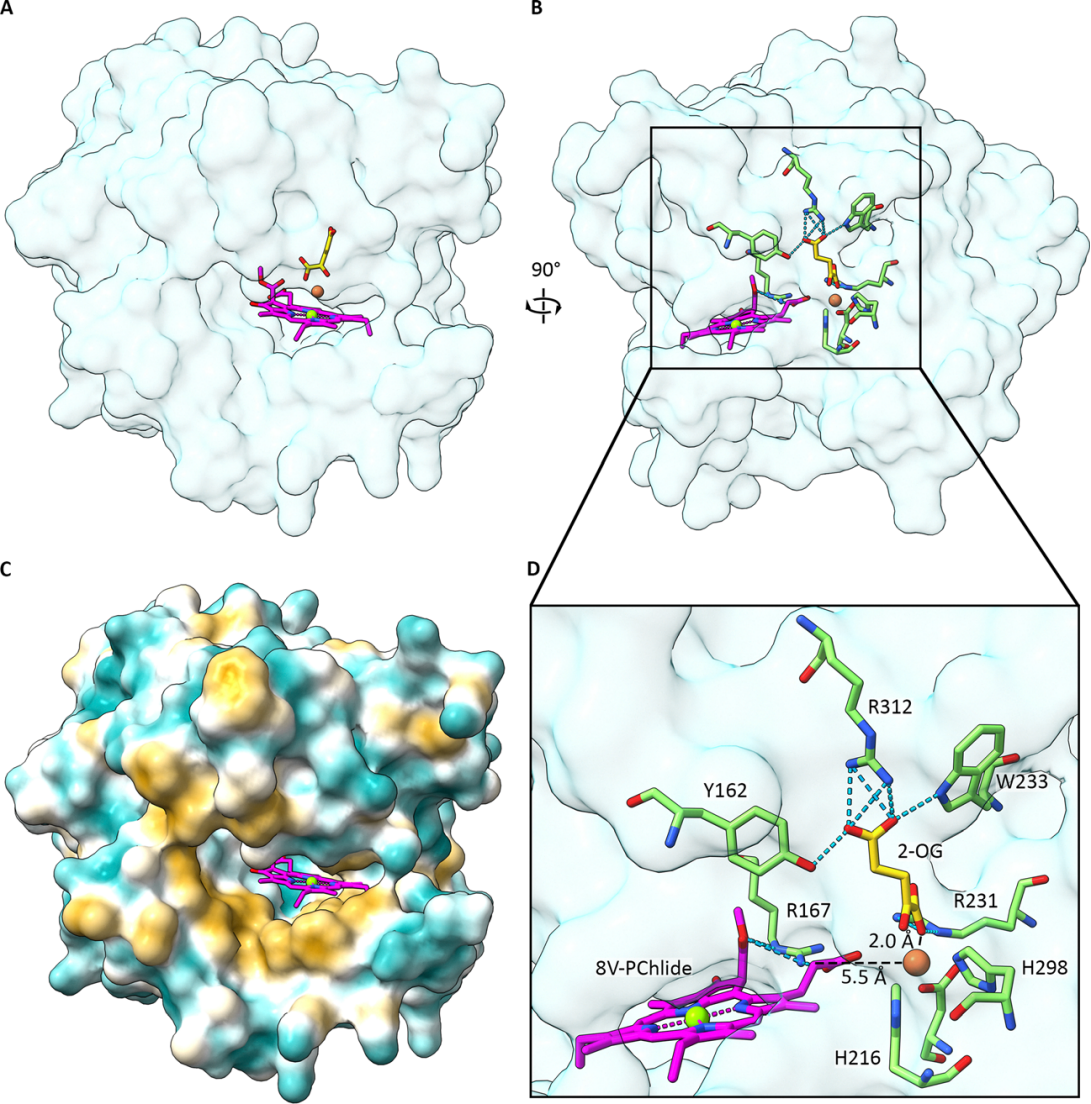
AF3 model of the *Phaeodactylum tricornutum* Chl *c* synthase, in a complex with its 8V-PChlide *a* substrate and its cofactors, 2-oxoglutarate (2OG) and Fe^2+^. (A) and (B) Two surface views of the overall topology of the protein with substrates and cofactors shown in stick representation. 8V-PChlide *a* is in magenta (heteroatoms coloured: blue – nitrogen, red – oxygen), 2-OG in yellow (heteroatom coloured: red – oxygen), and Fe^2+^ is shown in dark orange. (C) Surface hydrophobicity (gold – hydrophobic, blue – hydrophilic) showing an open substrate binding cleft for 8V-PChlide *a* flanked by hydrophobic residues. (D) Expanded view of the active site highlighting important residues. R167 is predicted to form hydrogen bond interactions with the C13^2^ methoxycarbonyl and the C17 propionate group. 2-OG binding is stabilised by several interacting residues: the C4 carbonyl group is predicted to form interactions with Y162, R312 and W233, while the C2 carbonyl group interacts with R231. The 2-keto group of OG and one of the C2 carbonyl oxygens provide coordination bonds to the Fe^2+^, along with D218, R231 and H298. The remaining coordination site remains free and pointing at the C17^2^ carbon, where oxidation occurs. The distance between the Fe^2+^ ion and the C17^2^ carbon is 5.5 Å. See Table S1 in the SI for ipTM scores for the AF3 model.

In the absence of a Chl *c* synthase structure, AF3 was used to calculate a model with its substrate and co-factors, 2-OG and Fe^2+^ (Fig. 12). R167 interacts with 8V-PChlide *a*, providing hydrogen bonds to the C13^2^ methoxycarbonyl and the C17 propionate carbonyl oxygen, which presumably orients the C17^2^ carbon for attack by a water coordinated by the Fe^2+^ ion. This Fe^2+^ is coordinated by H216, R231, H298 and the 2-keto and 2-carbonyl groups of 2-OG, which leaves a single coordination site unoccupied and pointing towards the C17^2^ carbon where oxidation occurs, only 5.5 Å from the Fe^2+^. It is feasible that an activated, nucleophilic water is held in this final coordination site and abstracts a proton from the C17^2^ carbon initiating the oxidation. The C4 carbonyl group of 2-OG is also predicted to interact with Y162, R312 and W233, which presumably act to orient the 2-OG correctly and stabilise binding (Fig. 12D). As with most other enzyme structures of the Chl pathway, a hydrophobic patch surrounding the substrate binding cleft was identified, suggesting its docking to the membrane surface for substrate retrieval and product release (Fig. 12C).

### 5.2. Biosynthesis of Chl *b*

Chl *b* helps to fill the 450–600 nm ‘green gap’ between the B band and Q_*Y*_ absorption features of Chl *a* (Fig. 2). This pigment differs from Chl *a* in having a C-7 formyl group and it is highly abundant globally, by virtue of its essential role in the major light-harvesting complexes of algae and plants. Conversion of the 7-methyl group to a 7-formyl is catalysed by Chl *a* oxygenase (CAO), first discovered in *C. reinhardtii*.^205^ This reaction requires molecular oxygen,^206,207^ and CAO has consensus sequences for a Rieske-type [2Fe–2S] cluster and for a mononuclear non-heme Fe-binding site. A study of the recombinant enzyme from *A. thaliana* required reduced Fd, replenished by FNR and an NADPH-regenerating system. It was found that CAO was specific for Chlide *a* rather than Chl *a*, and that the formation of the 7-formyl group proceeded in two steps *via* a 7^1^-OH intermediate,^208^ the same transient metabolite found in the conversion of Chl *b* to Chl *a*.^209^ A later, detailed study with several recombinant enzymes showed they could catalyse the formation of Chlide *b* from Chlide *a*,^210^ so the CAO acronym should refer to Chlide *a* oxygenase, as originally proposed.^208^ This mechanistic work showed that CAO uses a novel Rieske chemistry to catalyse two successive monooxygenation reactions, and that CAO uses Rieske oxygenase chemistry to convert 7^1^-OH–Chlide *a* to Chlide *b*, establishing 7^1^-OH–Chlide *a* as a true intermediate of the CAO reaction.^210^

**Fig. 13:**
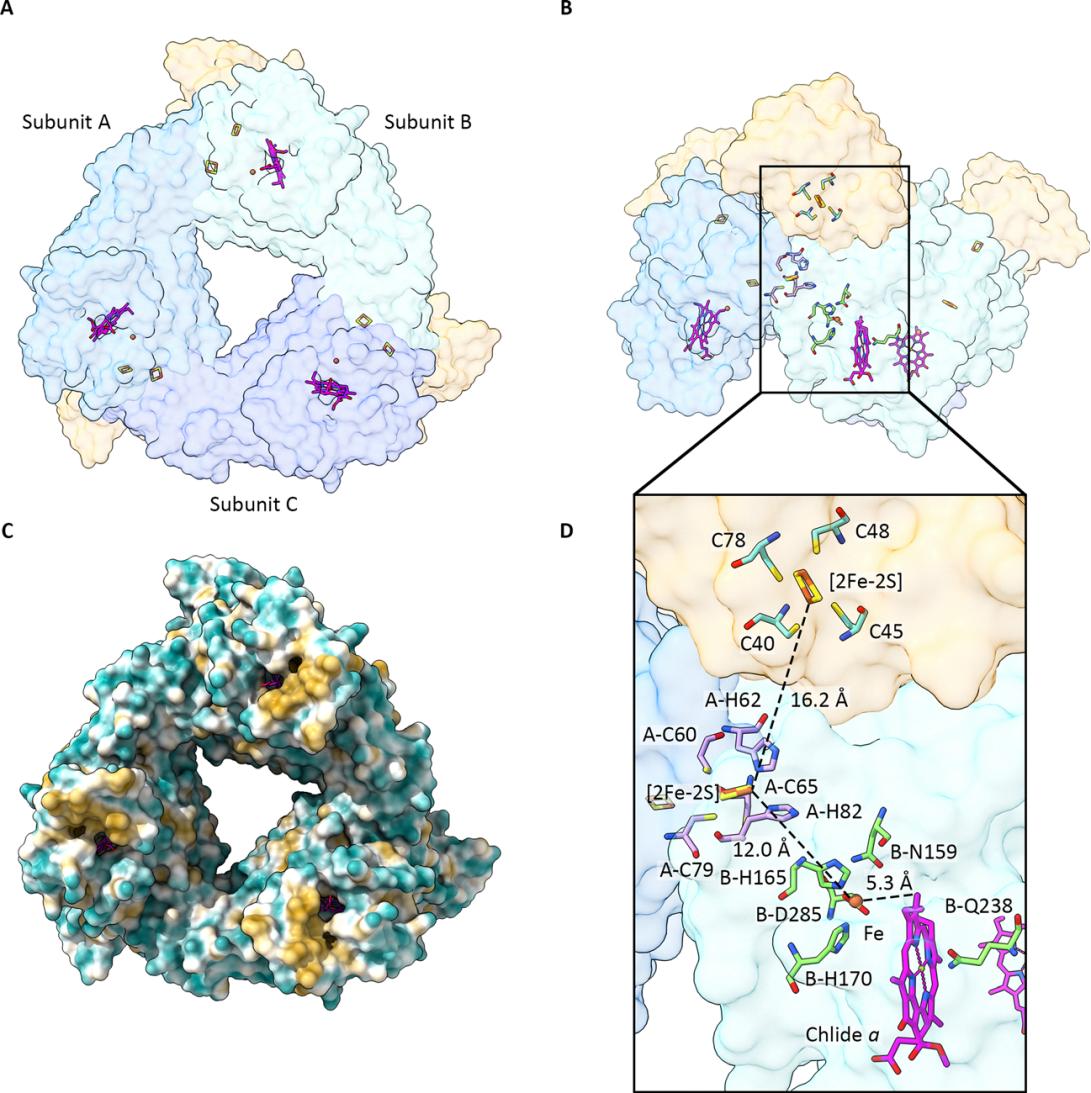
AF3 model of the trimeric chlorophyll(ide) *a* oxygenase (CAO) from *A. thaliana* (subunits coloured in light blue, blue and violet), in a complex with its Chlide *a* substrate, [2Fe–2S] and Fe^2+^ cofactors, and Fd (pastel orange), which has its own [2Fe–2S] cofactor. (A, B) Two surface views of the overall topology of the CAO trimer with substrates and cofactors shown in stick representation. Chlide *a* is in magenta (heteroatoms coloured: blue – nitrogen, red – oxygen); [2Fe–2S] clusters are coloured by heteroatom: dark orange – iron, yellow – sulfur; Fe^2+^ is shown in dark orange. (C) Surface hydrophobicity (gold – hydrophobic, blue – hydrophilic) showing three open substrate-binding clefts for Chlide *a* flanked by hydrophobic residues. (D) Expanded view of the active site, highlighting the inter-subunit chain of redox-active cofactors, where electrons are transferred from the [2Fe–2S] cluster of the docked Fd to a [2Fe–2S] cluster bound within subunit A of CAO, and subsequently to an Fe^2+^ ion held in CAO subunit B, where the Chlide *a* substrate is also bound. Important substrate- and cofactor-interacting residues are shown, and distances are indicated by black dashed lines. See Table S1 in the SI for ipTM scores for the AF3 model.

Although there is no structure for CAO oxygenase, deep learning-based methods have been used to predict the tertiary structures of CAO from *A. thaliana* and the Prasinophyte *Micromonas pusilla*, along with the Fd interaction site and the Chl *a* binding cavity.^211^ Here, we used AF3 to calculate the trimeric structure of CAO from *A. thaliana* with Fd, Chlide *a* and its [2Fe–2S] cluster and Fe^2+^ cofactors (Fig. 13). The AF3 model shows that the Fd-binding site is formed by the interface between two neighbouring CAO subunits, the geometry of which necessitates a trimeric structure (Fig. 13B). Further indications of this constraint are found in the complex redox chain formed at the interface between two subunits, where Fd provides electrons to a [2Fe–2S] cluster in subunit A, after which electrons are transferred to a mononuclear Fe^2+^ ion in subunit B, where the substrate binding site is located (Fig. 13D). The subunit A iron–sulfur cluster is located 16.2 Å from the Fd [2Fe–2S] cluster and is coordinated by A-C60, A-H62, A-C65 and A-C79. The mononuclear Fe^2+^ ion is located 12 Å from the subunit A iron–sulfur cluster and is coordinated by B-N159, B-H165, B-H170 and B-D285; the Fe^2+^ is ∼5 Å from the C7 methyl group of Chlide *a*, which is held transiently by a weak coordination bond with B-Q238. A further Fe^2+^ coordination site is likely occupied by molecular oxygen, which would be ∼3 Å from the C7 methyl group of Chlide *a*. Progressive reductions of this oxygen likely activate the molecule for oxidation of the C7 methyl to a formyl group (Fig. 13D). As with most Chl biosynthesis enzymes, each CAO monomer has a hydrophobic patch surrounding the substrate binding cleft (Fig. 13C).

### 5.3. Biosynthesis of Chls *d* and *f*

Although the extensive literature on these pigments cannot be covered here, there are many excellent reviews, such as,^36^ that provide a detailed account of Chls *d* and *f*. Briefly, ring A formyl groups in Chls *d* and *f* redshift Q_*Y*_ absorption in Et_2_O to 686 and 695 nm, respectively (Fig. 2), and organisms able to make these pigments gain access to new spectral niches enriched in far-red light.^8,36^ Chl *d* is the major pigment in the cyanobacterium *A. marina*^35^ but, despite extensive efforts, no Chl *d* synthase has been found. Labelling experiments have shown that the 3-formyl is derived from molecular oxygen;^212^ conversion of Chl *a* to Chl *d* has been observed with thiol reagents^213^ and a thiol protease,^214^ and could involve cysteine-containing proteins such as allophycocyanins.^215^ However, this part of the review on enzyme structures will concentrate on Chl *f*, as the Chl *f* synthase has been identified.

Following the earlier isolation of Chl *d* in 1943,^216^ the discovery of Chl *f* in 2010^37^ was a landmark in Chl research. This most redshifted Chl, with two exocyclic conjugated groups on ring A, was discovered in a complex cyanobacterial community that forms mats that cover marine stromatolites. These layered, rock-like structures house complex consortia of cyanobacteria resulting in high levels of spectral shading deep within the stromatolite.^217^ Culturing of samples from a stromatolite found in Shark Bay, Australia, under far-red light conditions, yielded a Chl *f*-containing filamentous cyanobacterium that was purified and named as *Halomicronema hongdechloris*.^218^ Gan *et al.*^219^ studied another cyanobacterium able to adapt to other niches enriched in far-red light, *Leptolyngbya* sp. strain JSC-1, and characterised the remodelling of the phycobilisome and photosystems as “far-red light photoacclimation” (FaRLiP). Far-red light activates a conserved cluster of 20 genes in *Leptolyngbya*, one of which, *psbA4*, encodes a functionally inactive (“super-rogue”) version of the PsbA subunit of PSII, identified previously in some diazotrophic bacteria,^220^ and also found in two other cyanobacteria capable of FaRLiP, namely *Chlorogloeopsis fritschii* PCC 9212 and *Synechococcus* sp. PCC 7335. Heterologous expression of the *C. fritschii psbA4* gene in *Synechococcus* sp. PCC 7002 conferred the ability to synthesise Chl *f*, showing that *psbA4* encodes at least part of the Chl *f* synthase.^221^

Unlike the conversion of the C7 methyl to a formyl, catalysed by CAO (see Section 5.2), the oxidation of the Chl *a* C2 methyl group to a formyl in Chl *f* is light-induced, and the Chl *f* synthase (ChlF) is a photo-oxidoreductase.^221^ ChlF lacks most of the ligands for binding the water-oxidising Mn_4_Ca_1_O_5_ cluster but it has Chl *a*, pheophytin *a*, tyrosine Y_Z_, plastoquinone and binds carotenoids. Heterologous production and purification of [His]_10_-tagged ChlF from *Fischerella thermalis* PCC 7521 in a Δ*psbD1* Δ*psbD2* strain of *Synechococcus* sp. PCC 7002 yielded 3–4% Chl *f* relative to total Chl content, about half the content of FaRLiP strains grown in FRL.^222^ ChlF activity was inhibited by DCMU, consistent with plastoquinone as an electron acceptor, and alteration of the putative Chl_*Z*_, P680 and Q_A_ binding sites inactivated the synthase but activity was unaffected by donor side electron transfer mutants.^222^ The Chl_Z_ pigment was proposed as a candidate for oxidation to Chl *f*.^223^

Parallel studies were conducted on *Synechocystis* harbouring FLAG-tagged ChlF from *Chroococcidiopsis thermalis* PCC 7203 or *F. thermalis*.^224,225^ An active PSII-like core complex was purified, termed the “super-rogue” PSII (srPSII) complex, which contained mainly ChlF (srD1), D2, CP47 and CP43, and enhanced expression of the *C. thermalis chlf* increased production of Chl *f* pigment 30-fold, reaching 8.2% Chl *f*/Chl *a*, a level comparable with that accumulated during FaRLiP acclimation.^225^ M127–G128 in the *Synechocystis* PSII subunit D1 were replaced by the QD motif, which is conserved across ChlF srD1 proteins, and this small change enabled the production of 0.1% Chl *f*/Chl *a*, demonstrating the likely role of this motif in Chl *f* production.^224^ In one suggested mechanism for the oxidation of the Chl *a* C2 methyl group to a formyl, the QD motif lies 13 Å from the proposed site for generating reactive oxygen species (ROS), namely the pheophytin *a* in the Chl *f* synthase corresponding to the redox-active electron acceptor in PSII.^224^

**Fig. 14:**
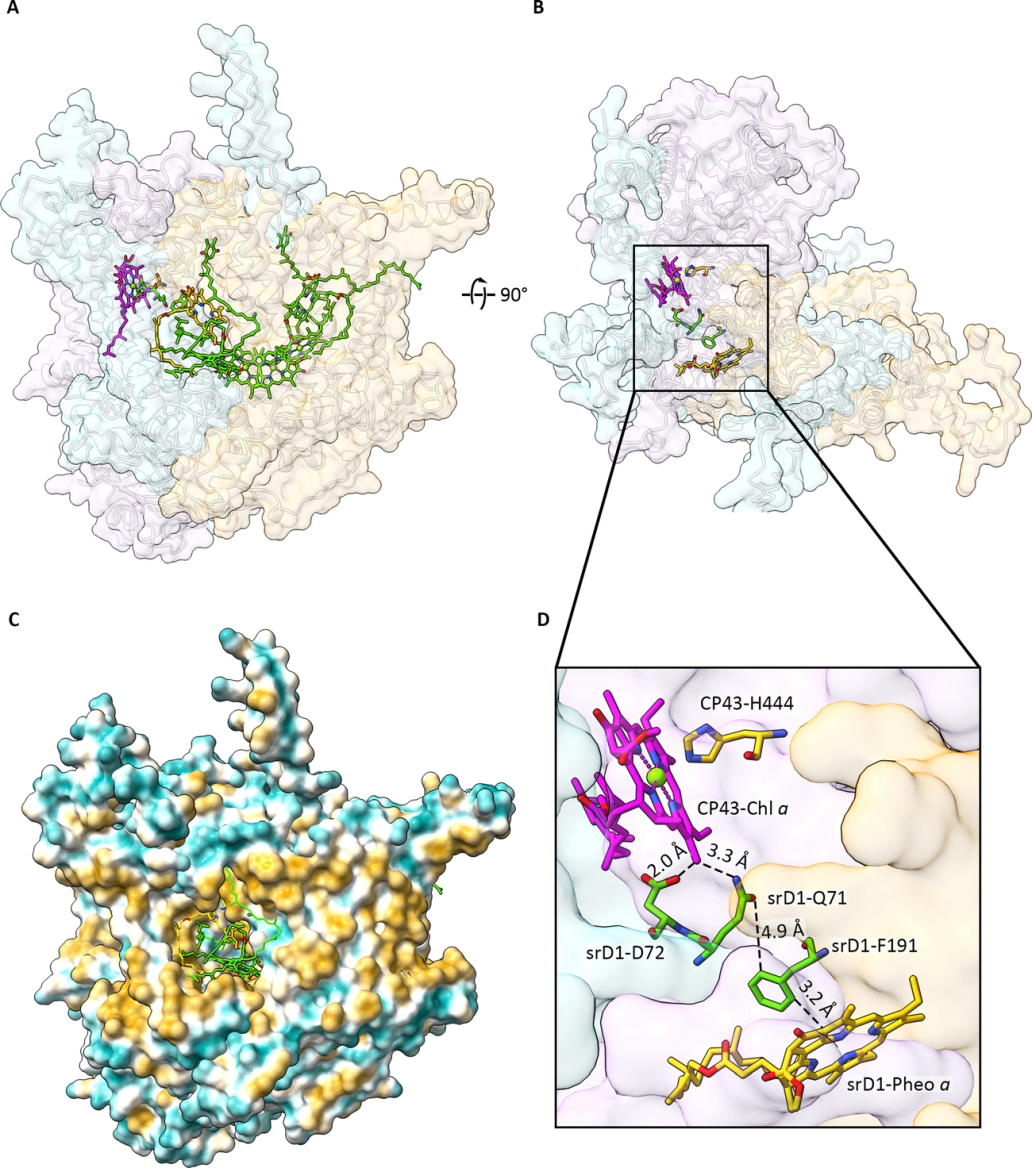
AF3 model of the srPSII Chl *f* synthase composed of srD1 (light blue), D2 (pastel orange) and CP43 (light pink) with its co-factors P680, ChlD1, ChlD2, PheoD1, PheoD2, two PQ-9 molecules and beta-carotene (all shown in green, stick representation with heteroatoms coloured: blue – nitrogen, red – oxygen). Also shown is the proposed substrate, a CP43-bound Chl *a* (magenta, stick representation with heteroatoms coloured: blue – nitrogen, red – oxygen). (A) and (B) Two surface views of the overall topology of the Chl *f* synthase with transmembrane helixes shown in cartoon representation in light grey. (C) Illustration of the transmembrane nature of the synthase showing the surface hydrophobicity (gold – hydrophobic, blue – hydrophilic). (D) Residues relevant to the function of the srPSII Chl *f* synthase enzyme, with distances indicated using black dashed lines. See Table S1 in the SI for ipTM scores for the AF3 model and RMSD values for the correspondence of models with any experimentally determined structures in the RCSB PDB.

Here, we used AF3 to generate a structural model of Chl *f* synthase based on ChlF/srD1 from *Synechoccocus* sp. PCC 7335, which includes CP43 (light pink), srD1 and D2 (Fig. 14). All the Chls and carotenoids for CP43 were modelled but are not shown. The srPSII complex, comprising srD1 (light blue) and D2 (pastel orange), is shown with most of the usual PSII co-factors highlighted in green, except for the proposed catalytic srD1–pheophytin *a*, which is shown in yellow (Fig. 14A). Following initial charge separation in the Chl *f* synthase, the reduced pheophytin (srD1–Pheo *a* in Fig. 14D) transfers an electron to a nearby oxygen generating a catalytically required ROS. SrD1–F191 is 3.2 Å from srD1–Pheo *a* and 4.9 Å from the QD motif, and may mediate electron transfer from the pheophytin to molecular oxygen (not shown) bound near the C2 methyl group of the Chl *a* shown in magenta in Fig. 14D. This Chl, the proposed substrate of the Chl *f* synthase, forms a ligand to CP43 at the interface of the CP43–srD1 complex. The role of the QD motif is unclear but it may stabilise a transition state on the way to oxidation of the C2 methyl group to a formyl. Alternatively, it could stabilise the ROS such that oxidation of the C2 methyl group is favoured.

The discovery of Chl *f* has had a significant impact on photosynthesis research, from its role in antenna complexes,^226,227^ primary photochemistry;^228,229^ and its possible future contribution to enhancing crop productivity.^230,231^ Thus, there has been a lot of interest in conferring the ability to make Chl *f* on plants, so they can use more >700 nm light for photosynthesis, but this requires more knowledge of the Chl *f* synthase. If Chl *a* is the substrate, Chl *f* synthase could lie in proximity to Chl synthase and the geranylgeranyl reductase ChlP, ready to accept their Chl *a* product. Another possibility is that the true substrate for the Chl *f* synthase is Chlide *a*, and the Chlide *f* product would require coupling to ChlG and ChlP for attachment and reduction of GGPP. Co-location of the Chl *f* synthase with other membrane-attached and membrane-embedded Chl biosynthesis enzymes is likely to be important, as would integration with the machinery for photosystem assembly.^191^ Then, heterologously produced Chl *f* must finally be incorporated into photosystems, replacing some native Chl *a* pigments, as already observed for hybrid PSI complexes in a strain of *Synechococcus* sp. PCC 7002 that expresses the Chl *f* synthase gene.^232^

## 6. Transfer of substrates and products between chlorophyll biosynthesis enzymes

The biosynthetic intermediates in the Chl pathway absorb light and are potentially photolabile, with the capacity to generate harmful ROS. Yet Chl biosynthesis and the assembly of Chls into active photosystems, as well as the repair of damaged complexes, must take place in the glare of the Sun. These considerations necessitate the rapid transfer of metabolites between enzymes, so the final Chl product can be efficiently assimilated into photosystem complexes where carotenoids can provide some measure of photoprotection. Perhaps the Chl synthase, coupled as it is to the membrane assembly machinery,^191^ is especially vulnerable, hence the complex formed with carotenoid-binding Hlips.^187^ One way to achieve efficient transfers of substrates and products between enzymes would be to ‘hardwire’ the whole pathway together *via* a set of specific protein–protein associations to form a Chl biosynthesis ‘supercomplex’; this level of organisation would promote substrate channelling between enzymes and prevent the intermediates from diffusing randomly (and damagingly slowly) to find their cognate pathway enzymes.

The availability of a complete set of Chl enzyme structures, some of which are in the PDB but here are all predicted by AF3, suggests another possibility; as noted in the previous section, five of the seven pathway enzymes (methytransferase, cyclase, POR, 8VR, GG reductase; Fig. 6C, 7C, 8C, 9C and 11D) appear to have a pore or active site cleft flanked by hydrophobic residues, which would allow each enzyme to sit on the membrane. The same features were found for the Chl *c* synthase (Fig. 12C) and CAO enzymes (Fig. 13C). Thus, Chl pathway intermediates could migrate between enzymes, and gain access to active sites, *via* the underlying membrane, which acts as a quasi-two-dimensional solvent. In the case of the membrane-intrinsic Chl synthase (Fig. 10D) and Chl *f* synthase (Fig. 14C), the active site clefts are accessed directly from within the bilayer. Thus, among our AF3 structures, MgCh is the only outlier, although a positively charged patch on ChlH could allow interaction of the MgCh complex with the negatively charged thylakoid membrane (see Section 4.1.3).

**Fig. 15:**
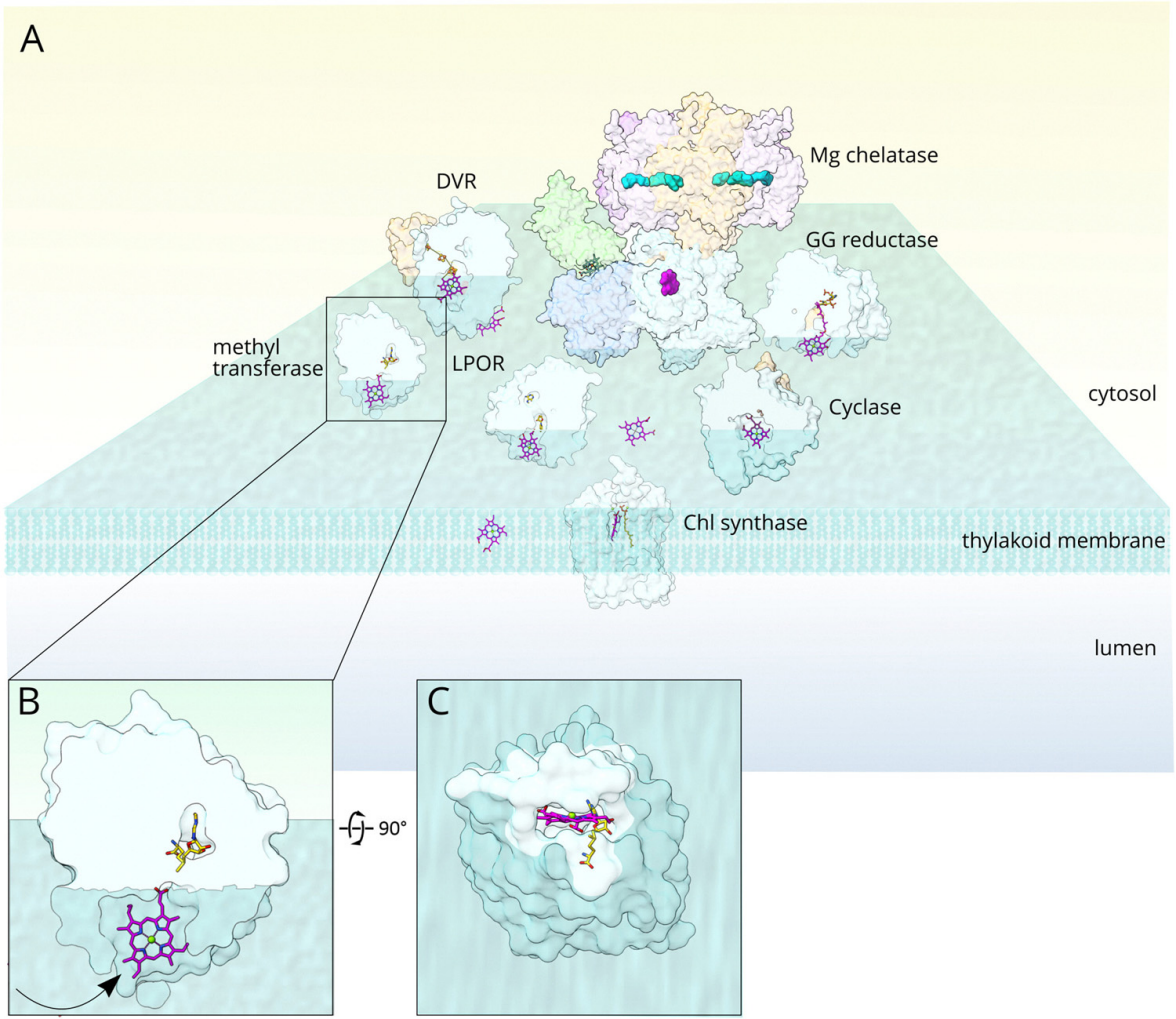
(A) Cartoon depiction of the membrane location and transfer of substrates and products between membrane-associated Chl biosynthesis enzymes. With the exception of MgCh, a slice through each enzyme structure shows that its active site cavity is flanked by hydrophobic residues that form a membrane interface, consistent with images of surface hydrophobicity in Fig. 6C, 7C, 8C, 9C and 11D. The extent of penetration of the Chl enzymes into the bilayer is purely to illustrate the proposal that Chl intermediates, represented by generic macrocycles in magenta, could gain access to, and move between, pathway enzymes *via* the membrane bilayer. (B) Zoomed view of the methyltransferase, used as an example of a Chl biosynthesis enzyme sitting in, but not through, the membrane. The direction of movement of the substrate into the active site cleft is shown with an arrow. (C) View of the methyltransferase from within the membrane, looking up at the membrane-embedded active site cleft.

We note that bilayer-forming lipids comprise only about half of the thylakoid membrane, with the remainder consisting of the non-bilayer lipid monogalactosyldiacylglycerol (MGDG); however, dense packing of membrane-intrinsic proteins such as photosystems can enforce local bilayer formation.^233^ It has been proposed that MGDG could create partially autonomous membrane domains,^233^ and perhaps one of these regions could encompass or house a Chl biosynthesis pathway. Fig. 15 depicts a cluster of membrane-associated Chl biosynthesis enzymes, but this model requires no particular organisation of enzymes, only their proximity. Communication between Chl biosynthesis components is proposed to rely on their shared location, forming a nanodomain on the membrane. If the pathway enzymes associate loosely to occupy 400 nm^2^ of membrane surface, roughly equivalent to a membrane volume of 2000 nm^3^, then one molecule of a biosynthetic intermediate has an effective concentration of approximately 0.8 mM. The kinetic parameters for these enzymes have been measured *in vitro* in the bulk phase, generally yielding *K*_d_ values in the 1–5 µM range,^79–81,102,110,111,130,155^ so with the minimal assumptions used here the effective intramembrane concentrations of Chl biosynthesis intermediates are much higher than their binding site affinities, thereby promoting formation of enzyme–substrate complexes. Such concentrations also greatly exceed the *K*_d_ values for enzyme–product complexes; for example, the *K*_d_ values for the porphyrin substrate and Mg–porphyrin product binding to ChlH do not differ much and are 4.0 and 5.2 µM, respectively.^81^ If this is the case, release of a product from the active site into the membrane bilayer will be more likely when the next enzyme in the pathway is ready to sequester this Chl intermediate within its own active site.

However, rather than considering a bulk parameter such as *K*_d_ the Chl pathway (and likely others), is best thought of as a series of single molecule encounters governed by a set of probabilities, where each Chl intermediate engages in a form of intermittent searching^234^ for its target, namely the next enzyme in the pathway. Each product transfers from its active site to the membrane, where it could randomly encounter up to five pathway enzymes with mostly unsuccessful (less probable) attempts at binding before finding its correct (most probable) active site. This intermittent search strategy is made easier if the enzyme targets are clustered in patches, and if the search is confined to two dimensions,^234^ which are both likely for the Chl pathway. Thus, the searching time is minimised, and although the diffusion rates for Chl intermediates in membranes are not known, a large molecule such as ubiquinone-10 (C_59_H_90_O_4_) can diffuse between photosystem and cytochrome complexes in milliseconds.^235^ Given the slow catalytic rates for some of the Chl pathway enzymes, such as 0.9 min^−1^ for the cyclase,^130^ Chl intermediates engaged in rapid, local searching for active sites would likely not limit the operation of the Chl pathway. There are useful regulatory consequences for this mechanism: the chain of product release/searching/active site binding events would rapidly come to a halt if one or more active target sites are already occupied and so unavailable to bind Chl intermediates. Chl enzymes would be locked in a product binding state, highly unlikely to discharge their products into the membrane because a product molecule is already in the vicinity (see the earlier point regarding *K*_d_ values for ‘free’ products exceeding those for enzyme–product complexes). Thus, a failure to complete Chl–protein assembly feeds back to Chl synthase because of the link between the synthase and the membrane insertase,^191^ and then product inhibition is propagated back along the series of Chl enzymes in the nanodomain *via* a series of single molecule encounters, shutting down the Chl pathway and preventing the accumulation of toxic Chl intermediates. Such a mechanism might explain why there is cessation of the BChl pathway in mutants of *Rba. sphaeroides* lacking genes encoding BChl–binding proteins,^236^ and no accumulation of pathway intermediates.

### 6.1. Consequences of the membrane transfer model for engineering hybrid biosynthetic pathways

Given the proposed lack of specific contacts between the Chl enzymes, it should be possible to assemble hybrid (B)Chl pathways from components obtained from a variety of bacteria, or from plant and bacterial sources. As long as the enzymes can congregate at a membrane surface, metabolites should be able to diffuse between them through the membrane bilayer. There are several examples of engineering native (B)Chl pathways, and of installing pathways *de novo* in purely heterotrophic bacteria, all of which rely on metabolites moving between (B)Chl enzymes in non-native contexts. *Rba. sphaeroides* has been used as a chassis to demonstrate the function of Chlide oxidoreductases (CORs) from *Rhodopseudomonas palustris*, *Cba. tepidum*, and *Roseiflexus castenholzii*.^237^ The native BChl *a* pathway of *Rba. sphaeroides* has been modified to synthesise BChl *b* by replacing the native COR genes with those from *Blastochloris viridis*;^238^ further modification of the *Rba. sphaeroides* isoprenoid biosynthetic pathway, and replacing the native BChl synthase with its counterpart from *H. modesticaldum*, yielded BChl *g* esterified with farnesol (BChl*g*_F_).^239^ Expressing *chlP* and *chlG* from *Synechocystis* in *Rba. sphaeroides*, re-routes the native BChl *a* pathway to Chl *a*.^240^ The oxidative MgPME cyclases can be swapped between *Rvi. gelatinosus* and *Synechocystis*, and they still function.^118^ Extending this work, the MgPME cyclases from *Synechocystis* and *A. thaliana* integrate into the BChl pathway in *Rvi. gelatinosus*.^120^ An early example using an *E. coli* chassis functionally linked the MgCh and methyltransferase enzymes from *Synechocystis*,^241^ and complete hybrid Chl and BChl pathways could be assembled in *E. coli* using components from *Synechocystis*, *Rvi. gelatinosus* and *Rba. sphaeroides*.^14,120^ Finally, cyanobacterial and plant chlorophyll synthases function in *Rba. sphaeroides*,^194,242,243^ and algal and plant Chl synthases can replace the native enzyme in *Synechocystis*, integrating into the Chl pathway and allowing normal assembly and function of photosystems.^244^

These many examples show that heterologous (B)Chl pathways can be assembled, and as long as there is only one foreign component a pathway can apparently function efficiently. However, pathways with diverse components often perform poorly when they are assembled in a heterotrophic host. For example, Chl or BChl production in *E. coli* is numbered in thousands of molecules per cell rather than the native millions in a photosynthetic bacterium, and efforts should be made to assemble these pathways with the normal stoichiometries and cellular levels of enzymes. Quantitative analysis of the *Synechocystis* Chl biosynthesis enzymes, in terms of copies per cell (cpc), yielded numbers in the 1150–3550 cpc range, with ChlI, Ycf54 and LPOR somewhat higher and 8VR and ChlG in the 250–1000 range.^245^ The stoichiometries of Chl biosynthesis enzymes revealed in that study, combined with their respective *k*_cat_ values (where available), highlight the potential for regulating various steps. For example, 500–1200 cpc of MgCh complexes might produce only 7–16 molecules of MgPIX s^−1^ cell^−1^, based on a *k*_cat_ of 0.013 s^−1^,^80,95^ whereas the methyltransferase has 10^5^ times more catalytic capacity on a cellular basis. As a result, there should be very little ‘free’ MgPIX. There are also mismatches between the very limited cyclase activity (1150–1700 cpc; *k*_cat_ of 0.015 s^−1^; 7–16 molecules MgPIX s^−1^ cell^−1^), and the 4–7-fold greater capacity of the next pathway enzyme, LPOR.^245^ Natively, photosynthetic cells have used these stoichiometries and (presumed) enzyme clustering to overcome the limitations of the enzyme chemistries and enzyme assembly, but pathways newly installed in a heterotrophic host effectively must start again, and there is much work to do before they can operate efficiently.

## Author contributions

CNH performed the literature search, prepared Fig. 1–4 and 15, wrote and edited the manuscript; FSM-B prepared Fig. 5–14, wrote and edited the manuscript.

## Conflicts of interest

There are no conflicts to declare.

## Supplementary Material

CB-007-D6CB00082G-s001

## Data Availability

The data supporting this article have been included as part of the supplementary information (SI). Supplementary information: Table S1. Structure files for AF3 models are available for download. See DOI: https://doi.org/10.1039/d6cb00082g.
