# Supplementary material for: The structural chemistry and biosynthesis of chlorophylls
Source: RSC Chem Biol. 2026 Jun 25;7(7):1134–66. doi: 10.1039/d6cb00082g (PMC13297055; doi:10.1039/d6cb00082g)
Supplement: CB-007-D6CB00082G-s001 [file CB-007-D6CB00082G-s001.pdf]

Supplementary Information (SI) for RSC Chemical Biology.
This journal is © The Royal Society of Chemistry 2026

Protein/complex	Fig.	ipTM* (protein-protein)	ipTM (protein-ligand)	Average ipTM	pTM**	RMSD values for AF3 vs structures in the RCSB PDB
**Mg chelatase**	5	ChlI-ChlD average: 0.69<br>ChlD-ChlH: 0.52<br>Gun4-ChlH: 0.88	ChlH-HEM: 0.7<br>ChlD-Mg: 0.65<br>ChlH-Mg: 0.7<br>Gun4-HEM: 0.58	0.59	0.62	Gun4: 0.368 Å (1Y6I)<br>ChlI: 0.959 Å (6L8D)<br>ChlH: 0.735 Å (6YSG)
**Mg PPIX O-methyl transferase**	6	N/A	ChlM-MgPPIX: 0.8<br>ChlM-SAM: 0.98	0.91	0.93	ChlM: 0.229 Å(4QDJ)
**Mg-PPIX monomethyl ester [oxidative] cyclase**	7	AcsF1-Ycf54: 0.92	AcsF1-μ-oxo-bridged-Fe: 0.98<br>AcsF1-MgPME: 0.91	0.92	0.91	Ycf54: 0.365 Å (5M2P)<br>PetF: 0.383 (5AUK)
**Light-dependent PChlide Oxidoreductase**	8	N/A	POR-NADPH: 0.97<br>POR-DVPChlide: 0.95	0.96	0.96	POR: 0.270 Å (6L1G)
**8-Vinyl reductase**	9	DVR-Fd: 0.96	Fd-[2Fe-2S]: 0.94<br>DVR-[4Fe-4S]-1: 0.98<br>DVR-[4Fe-4S]-2: 0.98<br>DVR-FADH: 0.97<br>DVR-DVChlide: 0.86			PetF: 0.383 (5AUK)
**Chlorophyll synthase**	10	N/A	ChlG-GGPP: 0.97<br>ChlG-Mg average: 0.95<br>ChlG-Chlide: 0.85			N/A
**GG-diphosphate reductase**	11	N/A	ChlP-Chl: 0.62<br>ChlP-NADPH: 0.93	0.8	0.85	N/A
**Chl c synthase**	12	N/A	ChlC-2OG: 0.98<br>ChlC-8V PChlide: 0.86<br>ChlC-Fe: 0.98	0.88	0.76***	N/A
**Chlorophyll(ide) *a* oxygenase**	13	CAO-Fd average: 0.73<br>CAO-CAO average: 0.94	Fd-[2Fe-2S] average: 0.89<br>CAO-Chlide average: 0.85<br>CAO-[2Fe-2S] average: 0.97<br>CAO-Fe average: 0.98	0.89	0.91	PetF: 0.383 (5AUK)
**Chl f synthase**	14		srD1-Chl: 0.72<br>CP43-Chl: 0.74<br>srD1-Pheo: 0.89	0.83	0.83	N/A

Table S1. Compilation of parameters for modelling Chl pathway enzymes. All structures are for the enzymes from *Synechocystis* PCC 6803, except for Chl *c* synthase from *Phaeodactylum tricornutum*, chlorophyll(ide) *a* oxidase (CAO) from *A. thaliana*, and Chl *f* synthase from *Synechoccocus* PCC 7335. The zipped structure files for all models are available.

*ipTM measures the accuracy of the predicted relative positions of the subunits forming the protein-protein complex. Values higher than 0.8 represent confident high-quality predictions, while values below 0.6 suggest likely a failed prediction. ipTM values between 0.6 and 0.8 are a grey zone where predictions could be correct or wrong. These values assume modelling with multiple recycling steps, so the process of prediction reaches a degree of convergence.

**pTM is an integrated measure of how well AlphaFold-Multimer has predicted the overall structure of the complex. It is the predicted TM score for a superposition between the predicted structure and the hypothetical true structure. A TM score above 0.5 means the overall predicted fold for the complex might be similar to the true structure. A TM score below 0.5 means the predicted structure is likely wrong.

*** Low pTM large, disordered section not shown in Fig 12 causing this low PTM

Information on ipTM and pTM available at https://www.ebi.ac.uk/training/online/courses/alphafold/inputs-and-outputs/evaluating-alphafolds-predicted-structures-using-confidence-scores/confidence-scores-in-alphafold-multimer/
